# Distinct signatures of gut microbiome and metabolites associated with significant fibrosis in non-obese NAFLD

**DOI:** 10.1038/s41467-020-18754-5

**Published:** 2020-10-05

**Authors:** Giljae Lee, Hyun Ju You, Jasmohan S. Bajaj, Sae Kyung Joo, Junsun Yu, Seoyeon Park, Hyena Kang, Jeong Hwan Park, Jung Ho Kim, Dong Hyeon Lee, Seonhwa Lee, Won Kim, GwangPyo Ko

**Affiliations:** 1 Department of Environmental Health Sciences, Graduate School of Public Health, Seoul National University, Seoul, 08826 Republic of Korea; 2 Institute of Health and Environment, Seoul National University, Seoul, 08826 Republic of Korea; 3 Center for Human and Environmental Microbiome, Institute of Health and Environment, Seoul National University, Seoul, 08826 Republic of Korea; 4 Division of Gastroenterology, Hepatology and Nutrition, Virginia Commonwealth University and McGuire VA Medical Center, Richmond, VA 23249 USA; 5 Division of Gastroenterology and Hepatology, Department of Internal Medicine, Seoul National University College of Medicine, Seoul Metropolitan Government Boramae Medical Center, Seoul, 07061 Republic of Korea; 6 Department of Pathology, Seoul National University College of Medicine, Seoul Metropolitan Government Boramae Medical Center, Seoul, 07061 Republic of Korea; 7 Department of Bio-convergence Engineering, Korea University, Seoul, 02841 Republic of Korea; 8 KoBioLabs, Inc., Seoul, 08826 Republic of Korea; 9 Bio-MAX/N-Bio, Seoul National University, Seoul, 08826 Republic of Korea

**Keywords:** Microbiome, Diagnostic markers, Liver fibrosis

## Abstract

Nonalcoholic fatty liver disease (NAFLD) is associated with obesity but also found in non-obese individuals. Gut microbiome profiles of 171 Asians with biopsy-proven NAFLD and 31 non-NAFLD controls are analyzed using 16S rRNA sequencing; an independent Western cohort is used for external validation. Subjects are classified into three subgroups according to histological spectra of NAFLD or fibrosis severity. Significant alterations in microbiome diversity are observed according to fibrosis severity in non-obese, but not obese, subjects. *Ruminococcaceae* and *Veillonellaceae* are the main microbiota associated with fibrosis severity in non-obese subjects. Furthermore, stool bile acids and propionate are elevated, especially in non-obese subjects with significant fibrosis. Fibrosis-related *Ruminococcaceae* and *Veillonellaceae* species undergo metagenome sequencing, and four representative species are administered in three mouse NAFLD models to evaluate their effects on liver damage. This study provides the evidence for the role of the microbiome in the liver fibrosis pathogenesis, especially in non-obese subjects.

## Abstract (web summary)

Nonalcoholic fatty liver disease (NAFLD) is associated with obesity but also found in individuals without obesity. Here, gut microbiome analysis using a biopsy-proven NAFLD cohort reveal distinct signatures of microbiome-metabolites associated with significant fibrosis in patients with NAFLD without obesity.

## Introduction

Nonalcoholic fatty liver disease (NAFLD) is characterized by hepatic manifestations of metabolic disorders, ranging from simple steatosis to nonalcoholic steatohepatitis (NASH), an aggressive histological form, ultimately leading to advanced fibrosis and cirrhosis. The global prevalence of NAFLD is estimated to be 24–30% in most epidemiological studies and is increasing in parallel with obesity and metabolic syndrome^1^. Although NAFLD is usually linked to obesity, NAFLD also occurs in non-obese subjects presenting with pathological severities similar to those observed in obese NAFLD patients^2,3^. In both the West and the East, 3–30% of non-obese populations are consistently reported to have NAFLD, without considering different body mass index (BMI) cut-offs for defining obesity (≥25 in Asians vs. ≥30 in other ethnicities)^4^. Although visceral fat, dietary composition, and genetic factors can be associated with non-obese NAFLD^5^, additional studies considering other environmental factors are required to elucidate the pathogenesis of non-obese NAFLD.

Increased interest has recently focused on identifying and understanding the specific role of the intestinal microbiota in various metabolic diseases. Gut dysbiosis has been associated with a decrease in beneficial short-chain fatty acid (SCFA)-producing bacteria, changes in the composition of bile acids^6^, activation of immune responses against lipopolysaccharide (LPS)^7^, increased ethanol production by overgrowth of ethanol-producing bacteria^8,9^, and conversion of phosphatidylcholine to choline and trimethylamine^10^. Changes in the gut microbiota, which affect the gut-liver axis, are also associated with the progression of chronic liver disease and advanced fibrosis, such as NAFLD and cirrhosis^11–15^. However, the microbial taxa associated with disease severity and fibrosis stage are not consistent with those reported in previous NAFLD studies. This inconsistency could be due to the effects of regional differences^16^. However, differential underlying BMI status might also account for these inconsistent results.

Here, the aims of this study are to determine whether the histological severity of NAFLD is associated with stool microbial changes in a well-characterized, biopsy-proven Asian NAFLD cohort and to explore microbial markers for the diagnosis of non-obese NAFLD. The 16S rRNA gene amplicon sequencing with 202 subjects stratified by obese status shows specific and distinct changes in the gut microbial community according to fibrosis severity, especially in non-obese subjects. After adjustments for age, sex, BMI, diabetes mellitus, and cirrhosis using multivariate association with linear models (MaAsLin), *Ruminococcaceae* and *Veillonellaceae* are the main microbial taxa associated with significant fibrosis in non-obese subjects. Moreover, stool primary bile acids and propionate levels are significantly elevated in non-obese subjects with worsening fibrosis severity. In bacterial taxa-metabolites networks according to fibrosis severity, strong inverse interactions between *Veillonellaceae* and *Ruminococcaceae* are noticeable and observed only in non-obese NAFLD subjects, implying the potential of the microbiome-based marker to diagnose fibrosis in non-obese NAFLD. The microbiome–metabolite combination suggested in this study significantly enhances the diagnostic power with an improved AUC of 0.939 (0.584 in all subjects; 0.520 in obese subjects), which is higher than that of fibrosis 4 index in non-obese subjects. We further conduct metagenomic shotgun sequencing of stool samples collected from 38 non-obese subjects, and species of *Megamonas* and *Ruminococcus* are identified as representative fibrosis-related taxa consisting of *Veillonellaceae* and *Ruminococcaceae*.

The metagenome analysis also reveals the relationship between significantly downregulated microbial genes related to primary/secondary bile acid metabolism and increasing amounts of total conjugated bile acids and unconjugated primary bile acids in non-obese subjects with significant fibrosis. The protective or worsening effect of four strains of bacteria belonging to *Ruminococcaceae* and *Veillonellaceae* on liver damage is investigated using three different mouse NAFLD models. *Ruminococcus faecis* shows an alleviating effect on fibrosis including significantly regressed histological severity and decreased expression of fibrogenic genes (*Col1a1, Timp1*, and *a-SMA*). Taken together, the intestinal bacteria and related metabolites in the pathogenesis of non-obese NAFLD have the potential to be utilized as a fibrosis marker, as well as a therapeutic target.

## Results

### Baseline characteristics of human subjects

This study included 171 subjects with biopsy-proven NAFLD (NAFL, *n* = 88; NASH, *n* = 83) and 31 no-NAFLD controls. All of the study subjects were classified into two groups (non-obese, BMI < 25; obese, BMI ≥ 25), and each group was divided into three subgroups according to the histological spectra of NAFLD or fibrosis severity. Supplementary Tables 1 and 2 present the detailed characteristics of each group, including clinical, metabolic, biochemical, and histological profiles. Subjects with NASH or significant fibrosis (F2–4) had higher levels of aspartate aminotransferase (AST), alanine aminotransferase (ALT), and insulin resistance in both the obese and non-obese groups. Subjects with significant fibrosis had higher NAFLD activity scores and presented more severe liver histology in terms of the histological classification of NAFLD (Supplementary Table 3 and Supplementary Fig. 1). More detailed baseline characteristics in each fibrosis stage, including well-known NAFLD-associated genetic variants, such as *PNPLA3, TM6SF2*, *MBOAT7-TMC4*, and *SREBF-2*, are shown in Supplementary Table 4.

### Microbial community changes according to fibrosis severity are different in non-obese and obese NAFLD subjects

**Fig. 1:**
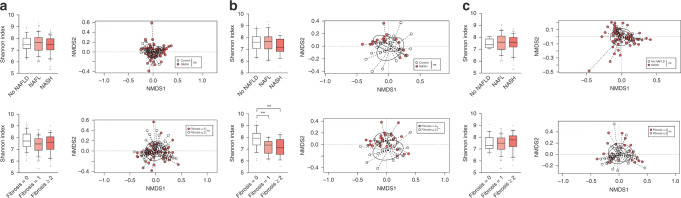
Comparison of the diversity of gut microbial communities in all, non-obese, and obese subjects. The alpha and beta diversity of **a** all (*n* = 202), **b** non-obese subjects (*n* = 64) (**P = 0.0074, F0 vs F1; **P = 0.0084, F0 vs F2–4), and **c** obese subjects (*n* = 138) divided by the histological spectra of NAFLD or fibrosis severity. Alpha diversity was based on the Shannon index with 12,000 rarefied sequences per sample. The box plots indicate the median, 25th to 75th percentiles (boxes), and 10th to 90th percentiles (whiskers). Statistical analysis was performed using a two-sided nonparametric Mann–Whitney test or a nonparametric Kruskal–Wallis test with Dunn’s multiple comparison test. NMDS plots were generated using relative OTU abundance data according to the Bray–Curtis distance, and statistical significance was measured using adonis analysis (panel **b**; *P = 0.038, F0 vs F2–4).

We analyzed data from 16S rRNA gene amplicon sequencing and compared the microbial diversity according to the histological spectra of NAFLD or fibrosis severity (Fig. 1). Alpha diversity based on the Shannon metric was plotted, and beta diversity based on the Bray–Curtis distance was plotted for comparison. No significant alterations in diversity among groups stratified by the histological spectra of NAFLD or fibrosis severity were observed (Fig. 1a). We then classified the subjects into two groups according to BMI status. In the non-obese group, a significant decrease in microbial diversity between F1 and F0 (*P* = 0.0074) as well as between F2–4 and F0 (*P* = 0.0084) was observed (Fig. 1b). Moreover, apparent clustering between F0 and F2–4 was observed (*P* = 0.038). In the obese group, no significant differences in diversity were found among groups stratified by the histological spectra of NAFLD or fibrosis severity (Fig. 1c). These results indicate that fibrosis severity rather than necroinflammatory activity is more likely associated with the changes of gut microbiome and underlying BMI status may also be an important factor responsible for alterations of the gut microbiome.

### Alterations of fibrosis-related microbiota are more prominent in non-obese NAFLD rather than obese NAFLD

**Fig. 2:**
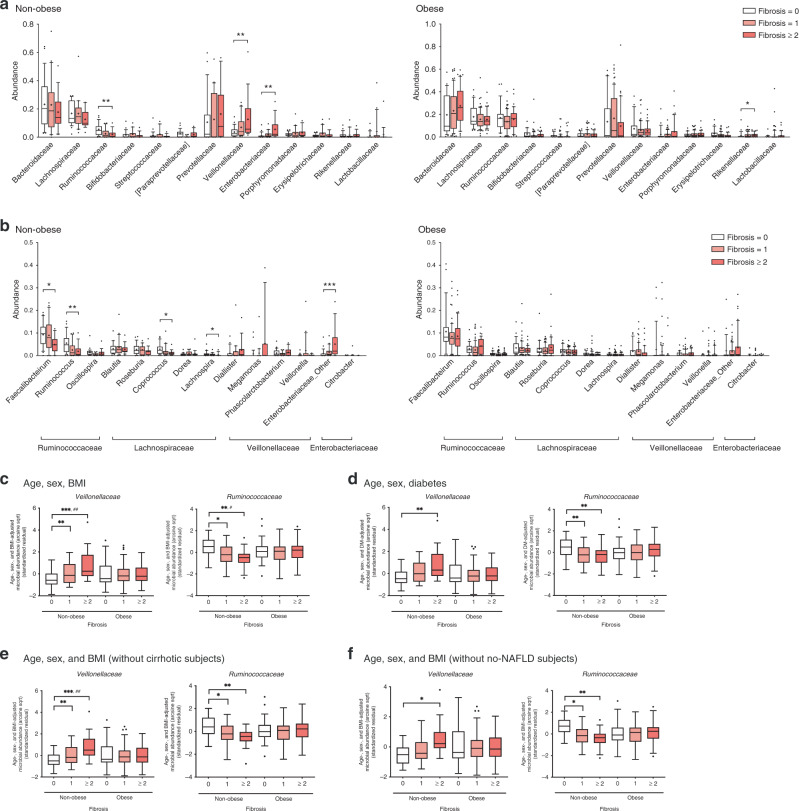
Phylogenetic comparisons of the gut microbiome in non-obese and obese subjects. **a** The 13 family- and **b** 14 genus-level taxa with top abundances are depicted for clarity. Statistical significance was measured using a nonparametric Kruskal–Wallis test with Dunn’s multiple comparison test. The *P* values are as follows: panel (**a**): **P = 0.0013, **P = 0.0054, **P = 0.0016, and *P = 0.04; panel (**b**): *P = 0.0217, **P = 0.0013, *P = 0.0209, *P = 0.0234, and ***P = 0.0014. Multivariate associations between specific gut-microbiome components and fibrosis severity stratified by obesity status (**c**–**f**). Arcsine-root transformed abundances of bacteria were regressed against **c** age, sex, and BMI, **d** age, sex, and diabetes, **e** age, sex, and BMI (without cirrhotic subjects), and **f** age, sex, and BMI (without no-NAFLD subjects). Statistical analyses for multivariate associations were performed using MaAsLin with adjustments for multiple comparisons (*q* value). The *P* and *q* values are as follows: panel (**c**): **P = 0.0057, ***P < 0.001, and ^##^*q* = 0.0297, *P = 0.0313, **P = 0.0012, and ^#^*q* = 0.0972; panel (**d**): from left, **P = 0.0020, **P < 0.0100, (F0 vs F1), and **P = 0.0013 (F0 vs F2–4); panel (**e**): **P = 0.0071, ***P = 0.0001, and ^##^*q* = 0.0339, *P = 0.0191, and **P = 0.0015; panel (**f**): *P = 0.0136, *P = 0.0292, and **P = 0.0023. The box plots indicate the median, 25th to 75th percentiles (boxes), and 10th to 90th percentile (whiskers). Colors inside the box represent fibrosis severity. **a**–**d** *n* = 64 for non-obese and *n* = 138 for obese; **e** *n* = 60 for non-obese and *n* = 125 for obese; **f** *n* = 43 for non-obese and *n* = 129 for obese. *P < 0.05, **P < 0.01, ***P < 0.001, ^#^*q* < 0.10, ^##^*q* < 0.05. Source data are provided as a Source Data file.

Differences in the specific microbial taxa by fibrosis severity in non-obese and obese subjects were compared using univariate and multivariate analyses (Fig. 2). In univariate analysis, the gradual enrichment of *Veillonellaceae*, as well as *Enterobacteriaceae*, was observed according to fibrosis severity in non-obese subjects (Fig. 2a). In obese subjects, *Rikenellaceae* was gradually enriched. In contrast, the abundance of *Ruminococcaceae* significantly decreased as fibrosis became more severe, which was observed only in non-obese subjects. At the genus level, *Faecalibacterium*, *Ruminococcus* (*Ruminococcaceae*), *Coprococcus*, and *Lachnospira* (*Lachnospiraceae*) were significantly depleted in the significant fibrosis group (Fig. 2b), while the abundances of *Enterobacteriaceae*_Other (*Enterobacteriaceae*) and *Citrobacter* gradually increased according to fibrosis severity. These alterations were also observed only in non-obese subjects. These results could be also found in the correlation plots (Supplementary Fig. 2). *Enterobacteriaceae* and *Veillonellaceae* had positive correlations (*P* = 1.09 × 10^−4^, *P* = 2.44 × 10^−3^, respectively), but *Ruminococcaceae* showed an inverse correlation (*P* = 4.41 × 10^−4^) with fibrosis severity.

For multivariate analysis, we adjusted for age, sex, and BMI using MaAsLin^17^. In the phylum Firmicutes, *Veillonellaceae* showed a steeper increase in relative abundance in non-obese subjects than in obese subjects (non-obese, *P* = 0.0002, *q* = 0.0297), while the abundance of *Ruminococcaceae* was inversely correlated with fibrosis severity in non-obese subjects (*P* = 0.0012, *q* = 0.0972) (Fig. 2c). *Ruminococcus*, a representative genus of *Ruminococcaceae*, also showed a significant inverse association with fibrosis severity only in non-obese subjects (*P* = 0.0011, *q* = 0.152) (Supplementary Fig. 3). In addition, *Veillonellaceae* and *Enterobacteriaceae* showed a significant, positive association with serum free fatty acid (FFA) levels in non-obese subjects (*q* = 0.178, *q* = 0.118, respectively) but not in obese subjects (Supplementary Fig. 4). Adipose tissue insulin resistance (adipo-IR) and glycosylated hemoglobin (HbA1c) were also positively correlated with the abundance of *Veillonellaceae* in non-obese subjects (adipo-IR, *q* = 0.142; HbA1c, *q* = 0.157). In contrast, serum FFA levels were inversely correlated with the abundance of *Ruminococcus* in all subjects (*q* = 0.0838) and in non-obese subjects (*q* = 0.144) but not in obese subjects (*q* = 1.00).

In addition to three variables (age, sex, and BMI), the presence of DM is also known to affect general changes in the microbial community^18^. We found that *Enterobacteriaceae* (*P* = 0.0002, *q* = 0.039) and *Faecalibacterium* (*P* = 0.0029, *q* = 0.12) were associated with the presence of DM in all subjects (Supplementary Fig. 5a). The depletion of *Lachnospira* (*P* = 4.36 × 10^−4^, *q* = 0.316) (Supplementary Fig. 5b) in the non-obese, as well as the enrichment of *Klebsiella* (*P* = 0.0026, *q* = 0.207) (Supplementary Fig. 5c), which belongs to *Enterobacteriaceae*, in the obese, was observed in subjects with DM. However, the associations of *Veillonellaceae* and *Ruminococcaceae* with fibrosis severity remained significant even after adjustment for DM (Fig. 2d) and the use of metformin (Supplementary Fig. 6). The anti-diabetic medications prescribed in each subgroup are listed in Supplementary Table 5. The oral hypoglycemic agents include metformin, linagliptin, glimepiride, etc., and some patients received combined treatment. To exclude the confounding effect of cirrhosis, we also performed sensitivity analysis only for study subjects without cirrhosis. Nonetheless, the significant associations of *Veillonellaceae* and *Ruminococcaceae* with fibrosis severity were not affected by cirrhosis (Fig. 2e). In addition, we conducted the same analysis with only NAFLD patients except for healthy control subjects. Similarly, *Veillonellaceae* and *Ruminococcaceae* were significantly associated with fibrosis severity notwithstanding the smaller population (Fig. 2f). To determine whether these notable microbiome changes in non-obese subjects could be attributed to the host gene effect, we adjusted for genetic variants of *PNPL3, TM6SF2, MBOAT7-TMC4*, and *SREBF-2* in multivariate analysis. The associations between two identified taxa and fibrosis severity in non-obese subjects remained significant even after adjustment for host genetic variants (Supplementary Fig. 7). Taken together, these findings indicate that enrichment of specific taxa according to fibrosis severity might be more prominent in the non-obese group than in the obese group.

### Non-obese and obese NAFLD subjects have different stool metabolites levels according to fibrosis severity

**Fig. 3:**
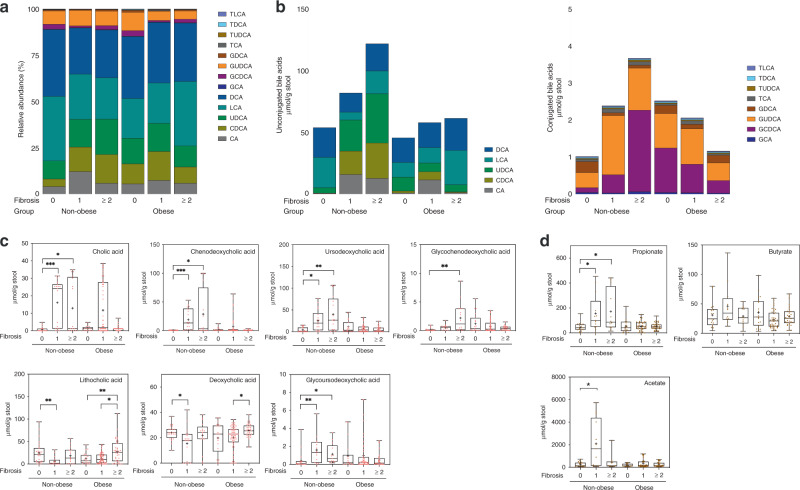
Comparison of stool metabolites levels stratified by fibrosis severity and obesity status. **a** Composition of bile acid profiles in different clinical settings. Stacked plots are generated using the average abundances of the 13 bile acids. **b** The stacked bars represent (left) unconjugated bile acids levels and (right) conjugated bile acids levels, which are stratified by fibrosis severity and obesity status. **c** Box plots represent the concentrations of the stool bile acids, which are stratified by fibrosis severity and obesity status; cholic acid (CA), ***P = 0.0007, *P = 0.0260, *n* = 57 for non-obese, and *n* = 111 for obese; chenodeoxycholic acid (CDCA), ***P < 0.0001, *P = 0.0164, *n* = 54 for non-obese, and *n* = 96 for obese; ursodeoxycholic acid (UDCA), *P = 0.0107, **P = 0.0012, *n* = 56 for non-obese, and *n* = 95 for obese; glycochenodeoxycholic acid (GCDCA), **P = 0.0023, *n* = 52 for non-obese, and *n* = 110 for obese; lithocholic acid (LCA), **P = 0.0075, *P = 0.0265, **P = 0.0045, *n* = 59 for non-obese, and *n* = 122; deoxycholic acid (DCA), *P = 0.0119, *P = 0.0128, *n* = 60 for non-obese, and *n* = 122 for obese; glycoursodeoxycholic acid (GUDCA), **P = 0.0064, *P = 0.0115, *n* = 47 for non-obese, and *n* = 99 for obese. **d** Box plots represent the most abundant fecal short-chain fatty acids (SCFAs) levels (acetate, propionate, and butyrate), which are stratified by fibrosis severity and obesity status; propionate, *P = 0.0114, *P = 0.0182, *n* = 51 for non-obese, and *n* = 100 for obese; butyrate, *n* = 43 for non-obese, and *n* = 98 for obese; acetate, *P = 0.0440, *n* = 42 for non-obese, and *n* = 89 for obese. The box plots indicate the median, 25th to 75th percentiles (boxes), and minimum to maximum values (whiskers). Outliers were removed by the ROUT method (*Q* = 1%) and data were analyzed using a nonparametric Kruskal–Wallis test with Dunn’s multiple comparison test. *P < 0.05, **P < 0.01, ***P < 0.001. Taurolithocholic acid (TLCA), taurodeoxycholic acid (TDCA), taurochenodeoxycholic acid (TUDCA), taurocholic acid (TCA), glycodeoxycholic acid (GDCA), glycocholic acid (GCA).

We next assessed the stool metabolites that are mainly associated with the gut microbiome using Q-TOF and GC-FID systems. The composition of the bile acid pool varied between non-obese and obese subjects (Fig. 3a and Supplementary Fig. 8). The levels of unconjugated and conjugated bile acids noticeably increased in non-obese subjects with fibrosis, and the total stool bile acid levels were higher (unconjugated bile acids, 2.3 times; conjugated bile acids, 3.6 times) in non-obese subjects with significant fibrosis (F2–4) than in those without fibrosis (F0) (Fig. 3b). In obese subjects, total conjugated bile acid levels decreased with increasing fibrosis severity. Specifically, cholic acid (CA), chenodeoxycholic acid (CDCA), ursodeoxycholic acid (UDCA), glycochenodeoxycholic acid (GCDCA), and glycoursodeoxycholic acid (GUDCA) levels significantly increased in non-obese subjects with worsening fibrosis severity (Fig. 3c). Lithocholic acid (LCA) and deoxycholic acid (DCA) levels were elevated in obese subjects with significant fibrosis, but only LCA reached statistical significance (Fig. 3c). Among three SCFAs, stool propionate levels gradually increased as fibrosis became more severe in non-obese subjects (non-obese; *P* = 0.0032, obese; *P* = 0.7979) (Fig. 3d).

### Non-obese NAFLD and obese NAFLD show different patterns of bacterial taxa-metabolites networks

To understand the interaction among gut microbiota in non-obese and obese subjects, the co-occurrence of taxa was measured and depicted with relative abundances in correlation with fibrosis severity (Supplementary Fig. 9). As expected, *Veillonellaceae* and *Enterobacteriaceae* were inversely correlated with *Ruminococcaceae* in non-obese subjects. However, strong interactions among these bacteria were not observed in the obese and all of the subjects, implying its specific role in the progression of fibrosis in non-obese subjects.

**Fig. 4:**
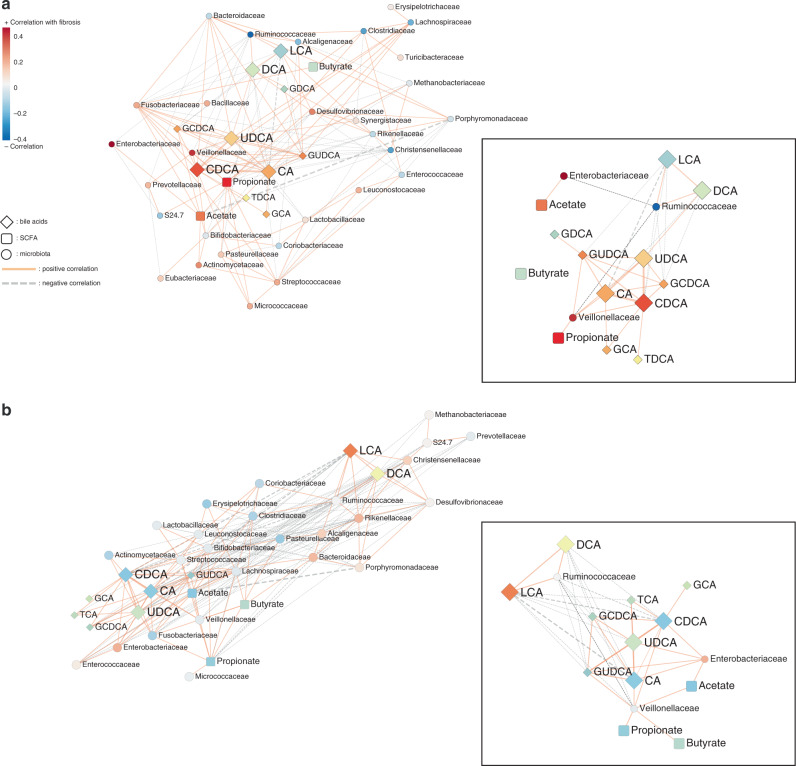
Network profiles between microbial taxa and stool metabolite components in non-obese and obese subjects. Co-occurrence coefficients among family-level microbiome components and stool metabolites were calculated by SparCC, and networks (*P* < 0.05) are depicted using Cytoscape. **a** Non-obese and **b** obese. The solid line (orange) and dotted line (gray) indicate positive and negative correlations, respectively. The shape of the node denotes the components used in this study (ellipse: microbiome, diamond: stool bile acids, and round rectangle: SCFAs) and the color indicates the degree of correlation with fibrosis severity. The *P*-value for each coefficient was obtained by bootstrapping the dataset 500 times and applying SparCC to each of those 500 datasets. Source data are provided as a Source Data file.

To explore the key drivers responsible for this observation, we analyzed the co-occurrence analysis of taxa and metabolites as illustrated in Fig. 4. The strong interactions among bile acids were observed in both non-obese and obese subjects (Fig. 4a and b). Interestingly, primary bile acids had an inverse relationship with *Ruminococcaceae*, which are known as indicators of healthy intestines, in both non-obese and obese subjects. *Veillonellaceae* showed positive interactions with primary bile acids and UDCA, as well as propionate. Bile acids usually retain the potential to modulate the growth of susceptible bacteria or enrich relatively resistant bacteria independent of obesity status. Nevertheless, the relationship of gut bacterial taxa and stool metabolites with severe fibrosis was more noticeable in non-obese NAFLD subjects than in obese NAFLD subjects.

### Microbiome–metabolite combination reflects significant fibrosis in non-obese subjects with NAFLD

**Fig. 5:**
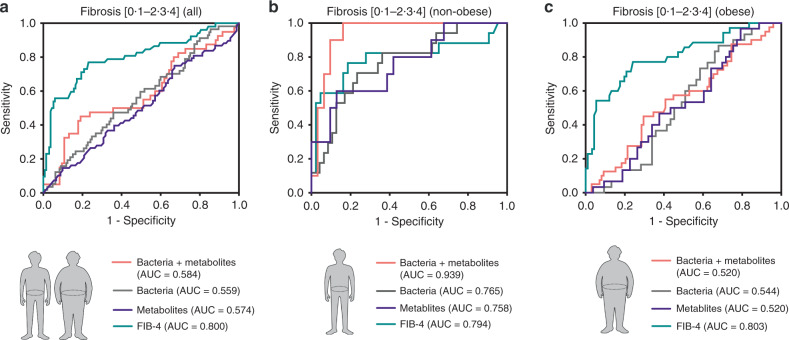
Receiver-operating characteristic (ROC) curves for the diagnosis of significant fibrosis in all, non-obese, and obese subjects. ROC curves using the combination of two bacteria (*Veillonellaceae* and *Ruminococcaceae*) and four stool metabolites (CA, CDCA, UDCA, and propionate) were plotted for the diagnosis of significant fibrosis in **a** all, **b** non-obese subjects, and **c** obese subjects, and the areas under the ROC curves (AUCs) were calculated. FIB-4, fibrosis 4 index.

To assess the utility of the gut microbiome for indicating significant fibrosis, the areas under the receiver-operating characteristic curve (AUCs) for diagnosing significant fibrosis were compared (Fig. 5). *Veillonellaceae* and *Ruminococcaceae* were selected as the most representative and significant fibrosis-related bacterial taxa. The combined bacterial marker to diagnose significant fibrosis yielded an AUC of 0.765 in non-obese subjects (0.559 in all subjects; 0.544 in obese subjects). In addition, we selected four stool metabolites (CA, CDCA, UDCA, and propionate) as fibrosis-related metabolites, and the combination of the four metabolites diagnosed significant fibrosis with an AUC of 0.758 in non-obese subjects (0.574 in all subjects; 0.520 in obese subjects). The addition of stool metabolites to these bacterial taxa significantly enhanced the diagnostic power with an improved AUC of 0.939 (0.584 in all subjects; 0.520 in obese subjects). The diagnostic power of this microbiome–metabolite combination was higher in non-obese subjects than that of FIB-4, which is widely used as a non-invasive fibrosis test in NAFLD. Considering the broad spectrum of pathological changes between healthy controls and cirrhotic subjects, we also assessed the AUCs restricted to study subjects without cirrhosis (Supplementary Fig. 10a) or without healthy controls (Supplementary Fig. 10b). Nevertheless, the AUCs using six combined markers sustained high diagnostic power in non-obese subjects (0.838 and 0.810 in the populations without cirrhotic subjects and healthy controls, respectively).

### The Western NAFLD cohort similarly shows different microbiome patterns between non-obese and obese subjects

**Fig. 6:**
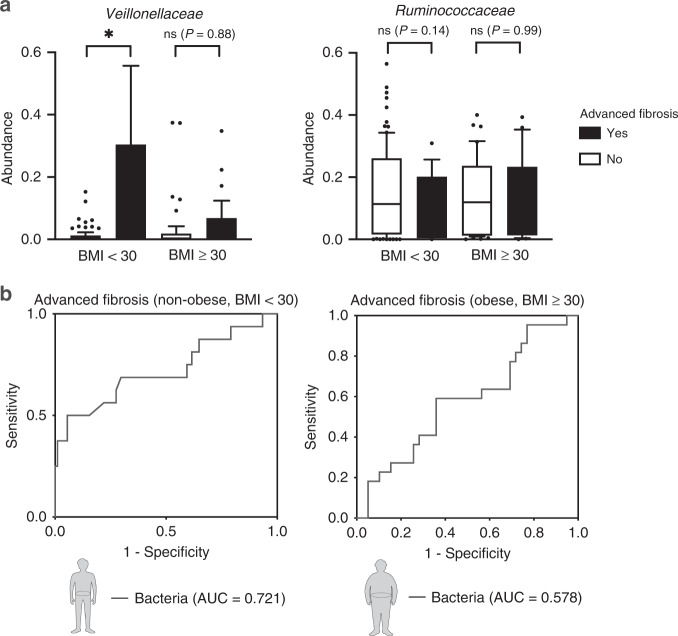
Validation of gut-microbiome differences between non-obese and obese subjects using a Western NAFLD cohort. **a** Abundance of *Veillonellaceae* and *Ruminococcaceae* stratified by obesity status and advanced fibrosis (*P = 0.0120, *n* = 107 for non-obese, and *n* = 61 for obese). **b** ROC curves using the combination of two selected bacteria (*Veillonellaceae* and *Ruminococcaceae*) were plotted for the diagnosis of advanced fibrosis in non-obese and obese subjects, and the areas under the ROC curve (AUCs) were calculated. The box plots indicate the median, 25th to 75th percentiles (boxes), and 10th to 90th percentiles (whiskers). Statistical analysis was performed using a two-sided Mann–Whitney test. *P < 0.05. ns, not significant.

To validate our results using an external independent cohort, we used public datasets. Caussy et al. reported a gut-microbiome-based biomarker for diagnosing NAFLD-related cirrhosis using a well-characterized NAFLD twin cohort^14^. A total of 168 subjects were divided into non-obese and obese groups according to BMI ($\ge$30, obese; <30, non-obese), and each group was divided based on the presence of advanced fibrosis. Baseline characteristics of the validation cohort are described in Supplementary Table 6. Non-obese subjects with advanced fibrosis were enriched with *Veillonellaceae* (*P* = 0.0120), which was not observed in obese subjects (*P* = 0.8818) (Fig. 6a). In addition, *Ruminococcaceae* showed a tendency to decrease only in non-obese (*P* = 0.1350; obese, *P* = 0.9944) subjects with advanced fibrosis. The combination of two fibrosis-related bacteria diagnosed advanced fibrosis with an AUC of 0.721 in non-obese subjects (0.578 in obese subjects) (Fig. 6b). Because the Western NAFLD twin cohort lacked metabolites data, the diagnostic power of the Western NAFLD twin cohort seemed to be lower than that of our Korean NAFLD cohort using the taxa-metabolite combination. These results indicate that changes in *Veillonellaceae*/*Ruminococcaceae* according to fibrosis severity in non-obese subjects are replicated in a Western cohort with the potential of the microbiome-based marker to diagnose fibrosis in non-obese subjects with NAFLD.

### Species of *Megamonas* and *Ruminococcus* are identified by metagenome sequencing

**Fig. 7:**
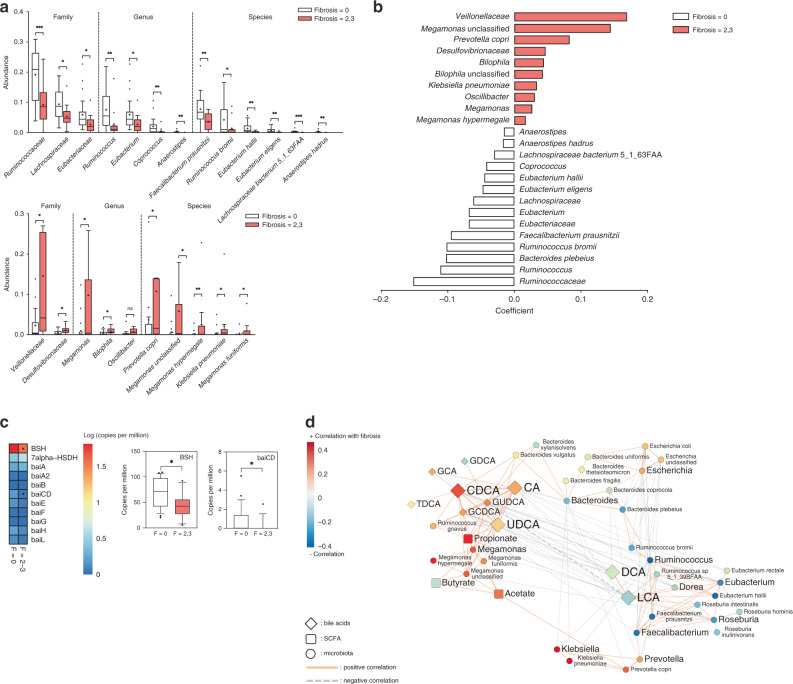
Identification of fibrosis-related bacteria in non-obese subjects using metagenomic shotgun analysis. Stool metagenome analysis of 38 non-obese subjects was conducted (F0, *n* = 25; F2–3, *n* = 13). **a** Abundances of fibrosis-related taxa are depicted for clarity. Taxa enriched in subjects with fibrosis stage 0 (top) and fibrosis stage of 2 or 3 (bottom). Statistical analysis was performed using a two-sided nonparametric Mann–Whitney test. The *P* values are as follows: (top) ***P = 0.0009, *P = 0.0113, *P = 0.0124, **P = 0.0024, *P = 0.0124, **P = 0.0051, **P = 0.0049, **P = 0.0070, *P = 0.0451, **P = 0.0041, **P = 0.0011, ***P < 0.0001, and **P = 0.0022; (bottom) *P = 0.0294, *P = 0.0346, *P = 0.0136, *P = 0.0163, *P = 0.0411, *P = 0.0164, **P = 0.0050, *P = 0.0112, and *P = 0.0336. **b** Multivariate associations between specific gut-microbiome components and fibrosis severity. Arcsine-root transformed abundances of bacteria were regressed against age, sex, and BMI. Only significant coefficients (*P* < 0.05) are depicted, and the color inside the box represents the enriched fibrosis group. **c** Heatmap showing the abundances of microbial genes related to bile acid metabolism pathway (left). The box plots indicate the median, 25th to 75th percentiles (boxes), and 10th to 90th percentiles (whiskers) (right) (*P = 0.0124 and *P = 0.0237). **d** Network profiles between microbial taxa and stool metabolite components. Coefficients among genus, species-level bacteria, and stool metabolite components were calculated by SparCC. Coefficients (*P* < 0.05) are depicted using Cytoscape. The *P*-value for each coefficient was obtained by bootstrapping the dataset 500 times and applying SparCC to each of those 500 datasets. The solid line (orange) and dotted line (gray) indicate positive and negative correlations, respectively. The shape of the node denotes the components used in this study (ellipse: microbiome, diamond: stool bile acids, and round rectangle: SCFAs) and the color indicates the degree of correlation with fibrosis severity. *P < 0.05, **P < 0.01, ***P < 0.001. Source data are provided as a Source data file.

To identify fibrosis-related species consisting of *Veillonellaceae* and *Ruminococcaceae*, metagenome analysis of stool samples collected from 38 non-obese subjects (F0, *n* = 25; F2–3, *n* = 13) was further conducted. Consistent with the results of 16S rRNA gene amplicon sequencing, *Veillonellaceae* and *Ruminococcaceae* were the most significant families related to fibrosis in both univariate and multivariate analyses of metagenome data (Fig. 7a, b). At the genus and species levels, *Ruminococcus bromii*, *Megamonas hypermegale*, and *M. funiformis* were the most significant taxa related to fibrosis severity in non-obese subjects. We also assessed the abundances of microbial genes related to primary and secondary bile acid metabolism (Fig. 7c). The abundances of genes encoding bile salt hydrolase (*bsh*) and 7α-hydroxy-3-oxochol-4-en-24-oyl-CoA dehydrogenase (*baiCD*) were significantly downregulated in non-obese subjects with significant fibrosis, which might be associated with increasing amounts of total conjugated bile acids and unconjugated primary bile acids. Major contributors to *bsh* were species belonging to *Ruminococcaceae*, *Lachnospiraceae*, and *Eubacteriaceae* (Supplementary Fig. 11).

The interaction between fibrosis-related bacterial species and stool metabolites was also revealed by network analysis based on the metagenome data of non-obese NAFLD subjects (Fig. 7d). The distinct co-occurrence pattern and strong correlation between key bacteria and metabolites were reconstructed; *R. bromii, Faecalibacterium prausnitzii*, and *Roseburia intestinalis* were inversely correlated with fibrosis severity and primary bile acid level, and *Megamonas* spp. showed a significant, positive correlation with primary bile species and UDCA, along with increasing fibrosis severity. However, *Ruminococcus gnavus*, a member of *Lachnospiraceae* family, was positively associated with primary bile acids and *Megamonas* species.

### Administration of *Ruminococcus faecis* alleviates liver damage in NAFLD mouse models

**Fig. 8:**
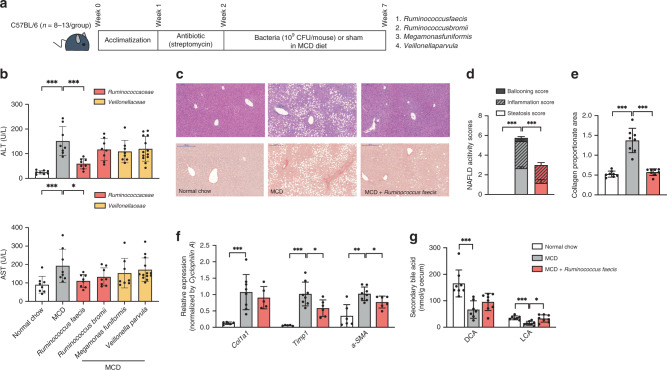
Effect of fibrosis-associated bacteria on liver damage induced by an MCD diet. Mice were acclimated for 1 week on a standard chow diet. Then, they were treated with streptomycin (1 g/L) in drinking water for colonization of four fibrosis-related bacteria. Following 5 weeks, the mice were given daily 200 μL of either bacteria (10^9^ CFU/mouse in PBS) or sham in an MCD diet. **a** Scheme of the animal experiment. **b** Effects of the MCD diet and bacteria on serum ALT and AST levels (ALT, ***P = 0.0002 and ***P = 0.0003; AST, ***P = 0.0047 and *P = 0.0281) *n* = 8 for normal chow, MCD, *R. faecis*, *R. bromii*, and *M. funifomis* group) and *n* = 13 for *V. parvula* group. **c** Representative images of *Ruminococcus faecis*-treated liver tissues stained with H&E and Sirius red. Scale bar indicates 200 μm. **d** Comparison of histological NAFLD activity scores calculated on H&E stained liver tissues (***P < 0.0001 and ***P = 0.0006; *n* = 12 for all groups). **e** Comparison of collagen proportionate areas measured on Sirius red-stained liver tissues (***P = 0.0002 and ***P = 0.0002; *n* = 8 for all groups). **f** Relative fibrogenic mRNA expression of liver harvested from *Ruminococcus faecis*-treated mice (**P = 0.0016, **P = 0.0016, *P = 0.0293, **P = 0.0047, and *P = 0.0356; *n* = 5–6 for *n*ormal chow and MCD + *Ruminococcus faecis*, *n* = 8 for MCD). **g** Comparison of secondary bile acids levels measured in the cecum of *Ruminococcus faecis*-treated mice (***P = 0.0003, ***P = 0.0006, and *P = 0.0104; *n* = 7–8 for *n*ormal chow and MCD, *n* = 8 for MCD + *Ruminococcus faecis*). The bar graphs indicate the means with SDs. Statistical analysis was performed using a nonparametric Kruskal–Wallis test with Dunn’s multiple comparison test or a two-sided Mann–Whitney test. *P < 0.05, **P < 0.01, ***P < 0.001.

To identify the protective or worsening effect on liver damage, we administered four representative species-level bacteria belonging to *Ruminococcaceae* and *Veillonellaceae–Ruminococcus faecis* (*R. faecis*), *R. bromii, Megamonas funiformis (M. funiformis)*, and *Veillonella parvula* (*V. parvula)* to C57BL/6 mice fed methionine- and choline-deficient (MCD) diets for 5 weeks (Fig. 8a). We demonstrated that *R. faecis* feeding decreased serum ALT and AST levels compared to sham feeding (Fig. 8b). No worsening effect was found in mice given *M. funiformis* and *V. parvula*. An alleviating effect of *R. faecis* on fibrosis was shown with H&E and Sirius red staining (Fig. 8c), and the histological severity of NAFLD induced by an MCD diet was significantly regressed in mice fed *R. faecis* (Fig. 8d, e). The mRNA expression of fibrogenic genes (*Col1a1, Timp1*, and *a-SMA*) was also downregulated in mice treated with *R. faecis* compared to untreated control mice fed an MCD diet (Fig. 8f). In parallel with the changes in biochemical and histological liver injury markers, the cecal levels of secondary bile acids (DCA and LCA) were also decreased by an MCD diet and increased by *R. faecis* treatment (Fig. 8g). To confirm the alleviating effect of *R. faecis* on liver damage in other mouse NAFLD models, we used a choline-deficient, L-amino acid-defined, high-fat diet (CDAHFD) model which prevents body weight loss in mice and shows no insulin resistance, and a genetic leptin-deficient (*db*/*db*) model, which develops spontaneously diabetes and fatty liver with insulin resistance. In both models, *R. faecis* administration decreased ALT and AST levels (Supplementary Fig. 12). However, the liver ratio against body weight decreased only in *db*/*db* mice. Nevertheless, the fasting insulin levels in serum and insulin resistance measured by ipGTT in *db*/*db* mice were not affected by *R. faecis* treatment.

In search of the relationship between administered taxa and changes in bile acid or SCFA composition, we further analyzed the compositions of bile acid and SCFA (Fig. 8g, Supplementary Fig. 13, and Supplementary Table 7). We observed that the cecal levels of LCA and DCA were decreased by an MCD diet and were normalized by *R. faecis* treatment. However, an increase in the cecal levels of SCFAs was not observed by *V. parvula* treatment, rather a slight decrease in the level of propionate was observed in the cecum. These results indicated that intestinal bacteria could affect the regression of NAFLD in an insulin-independent manner, supporting our human-associated data found in non-obese NAFLD subjects.

## Discussion

In our biopsy-proven Asian NAFLD cohort, we observed significant differences in the changes of gut microbiota according to fibrosis severity between non-obese and obese subjects. Interestingly, alterations in diversity with worsening fibrosis severity were observed only in non-obese subjects. Univariate and multivariate analyses using this cohort allowed us to focus on several microbes as potential targets to diagnose significant fibrosis. Furthermore, total bile acid levels, specifically primary bile acids and UDCA, and propionate levels in stool samples increased with worsening fibrosis severity in non-obese subjects. Utilizing these observations, we suggest that the combination of gut microbiota and stool metabolites might be a more robust diagnostic tool for diagnosing significant fibrosis in non-obese subjects with NAFLD. Moreover, the changes in fibrosis-related microbiota in non-obese subjects were validated using an independent Western NAFLD cohort. To confirm our association and demonstrate the causality, we performed metagenomic shotgun sequencing of 38 non-obese subjects and chose four bacterial species for animal validation. We administered four bacteria to MCD diet-induced NAFLD mice. *R. faecis* had a protective effect on liver damage in MCD diet-, CDAHFD diet-, and *db*/*db*-induced NAFLD mouse models.

In the current study, we used MaAsLin^17^ to identify NAFLD/fibrosis-specific bacteria. An important factor that is usually overlooked in microbiome studies is the removal of confounding effects, which can significantly influence microbiome data^17^. In addition, network analysis based on co-occurrence and microbial interactions supported distinct microbiome signatures in non-obese NAFLD patients identified by correlation and multivariate analyses. However, such notable microbiome changes were not related to host genetic traits that are known to be associated with NAFLD. The lack of the associations between genetic variants and bacterial taxa extends the previous study that reported the importance of environmental factors over genetic traits in shaping the human intestinal microbiota^19^.

Among three major fibrosis-related taxa, *Enterobacteriaceae* did not remain significant after adjustment for BMI, DM, and cirrhosis. *Enterobacteriaceae* was clustered by the presence of DM, which was consistent with the previous study^20^. Although *Enterobacteriaceae* was associated with DM and the use of anti-diabetic medications, the overgrowth of Proteobacteria, including *Enterobacteriaceae*, in severe liver disease has been commonly reported^11,15^.

The *Veillonellaceae* family, including six genera of Gram-negative bacteria (*Megasphaera*, *Veillonella*, *Dialister*, *Allisonella*, *Anaeroglobus*, and *Negativicoccus*)^21^, is known as a group of propionate-producing bacteria that utilize lactate as a substrate^22^. We first hypothesized that the enrichment of *Veillonellaceae* produces more propionates, which are absorbed into the liver, ultimately leading to the establishment of NAFLD. However, the administration of *M. funiformis* and *V. parvula* did not worsen liver damage and the cecal propionate levels were not increased in a mouse validation study. Taken together, the strong associations of *Veillonellaceae* with fibrosis severity and stool metabolites in our human studies were not attributed to the causal effects of the tested strains, but could be applied to a diagnostic marker for fibrosis in non-obese subjects with NAFLD.

*Veillonellaceae* has been associated with chronic liver disease, including cirrhosis and primary sclerosing cholangitis^14,15,23,24^. In an American cohort, Caussy et al. reported that *Megasphera* was enriched in a cirrhosis group^14^. In addition, Qin et al. reported that *Veillonella* was enriched in cirrhotic patients along with *Streptococcus*, in a Chinese cohort, and they hypothesized that altered bile acid production in cirrhotic patients enables *Veillonella* to originate from the mouth and colonize in the gut environment^15^. However, Bajaj et al.^13^ reported a decrease in *Veillonellaceae* in Western cirrhotic subjects (BMI 29.2–30.8) compared with BMI-matched healthy controls (BMI 23.9–35.1). The discrepancy in the enrichment of *Veillonellaceae* in cirrhotic subjects between previous studies might be attributed to several confounding factors in cirrhotic subjects such as varioius etiologies of cirrhosis, use of rifaximin and beta-blockers, the setting of decompensation, and other complications, including infections and metabolic/cardiovascular disorders. Therefore, we further evaluated and confirmed the significance of associations between the identified bacterial taxa and fibrosis severity and the diagnostic power of six markers in a population without cirrhotic subjects. Given the controversial results of association studies, the confounding effects of cirrhosis and other diseases, and the lack of a causal effect on fibrosis progression in the current study, *Veillonellaceae* could be used only as a diagnostic marker in a selected population, such as non-obese NAFLD patients.

In contrast, *Ruminococcaceae* acts as a key component of the microbiota in healthy people by maintaining homeostasis of the gut microenvironment^15^. *Ruminococcaceae* was gradually depleted with worsening fibrosis severity in combined and non-obese subjects with NAFLD, as also noted in the previous studies^9,11,13^. Two different studies compared gut-microbiome profiles according to obesity status in Latin American NASH and Asian NAFLD cohorts, respectively^25,26^. Similar to our results, the relative abundance of *Ruminococcus* was significantly reduced in non-obese NASH subjects compared to non-obese controls. However, Boursier et al. reported that the abundance of *Ruminococcus* was higher in subjects with significant fibrosis^12^. These discordant results could be partly attributed to the fact that Boursier et al. compared gut-microbiome profiles using only obese subjects (average BMI; 31), suggesting that non-obese subjects might have disparate gut-microbiome compositions compared to obese subjects.

Among the *Ruminococcaceae* family, *F. prausnitzii* was already reported to regulate the hepatic fat content and compositions of lipid species and to reduce adipose tissue inflammation in high-fat-fed mice^27^. To confirm the causal effect of other *Ruminococcus* genus on NAFLD, we administered *R. faecis* and *R. bromii* isolated from human stools^28^ in an MCD-diet-induced NAFLD model. *R. faecis* had a protective effect on liver damage, implicating the species specificity of *Ruminococcus* strains and that some intestinal bacteria have the potential to be utilized as a fibrosis marker, as well as a therapeutic target.

Herein, the synthesis of bile acids was elevated with worsening fibrosis severity, and it was observed only in non-obese subjects. Previous studies have also reported the elevated synthesis of bile acids in NAFLD patients^12,29^. Bile acids are crucial for the emulsification of lipids and for maintaining hepatic glucose homeostasis, and they exert a strong antimicrobial effect owing to their detergent properties, which likely change the environment of gut microbiota by inhibiting the growth of susceptible bacteria. Although bile acid tolerance shows a strain-specific feature, Gram-negative bacteria are generally more resistant to the antimicrobial effects of bile acids than Gram-positive bacteria^6,30^.

In the current study, *Ruminococcaceae* was depleted in non-obese subjects with significant fibrosis, suggesting the modulation of gut microbiome by altered bile acid composition. We also observed that microbial genes metabolizing bile acids were significantly altered in non-obese subjects with fibrosis. Notably, *bsh* and *baiCD* genes were downregulated and major contributors to the *bsh* gene were identified as species belonging to *Ruminococcaceae*, *Lachnospiracese*, and *Eubacteriaceae*.

In an MCD-diet-induced NAFLD model, 5-week *R. faecis* and *R. bromii* treatment increased the cecal levels of secondary bile acids, suggesting a potentially causal role of *Ruminococcus* in the synthesis of secondary bile acids in a direct or indirect way.

Sinha et al.^31^, reported that *Ruminococcaceae* were depleted and stool secondary bile acid levels were reduced in patients with ulcerative colitis, and that the supplementation of LCA ameliorated inflammation in the animal colitis model, implicating the potential therapeutic role of *Ruminococcaceae* in gut microenvironment.

We acknowledge several limitations of this study. First, the cross-sectional study allowed us to compare the stool microbiota and metabolites according to disease severity but did not explain the exact reason for changes in gut microbiota. Therefore, further longitudinal studies using a prospective non-obese NAFLD cohort are warranted to accurately predict non-obese NASH with significant fibrosis using the stool taxa-metabolite markers identified in the current study. Second, a substantial number of patients had DM in this cohort. Both NAFLD and DM are closely interrelated since they share insulin resistance as a common mechanism of pathogenesis^32^. Anti-diabetic medications such as metformin might exert a confounding effect on microbiome analysis, interfering with the discrimination of fibrosis-specific microbes. Although we adjusted for DM and anti-diabetic medications to distinguish the fibrosis-specific microbiome, further research using a non-diabetic NAFLD cohort is required. Third, the diagnosis and classification methods of fibrosis in the validation cohort (i.e., elastography) were different from those in our cohort (i.e., biopsy). Caussy et al. compared gut microbiota among the NAFLD-related cirrhosis, NAFLD without advanced fibrosis, and no-NAFLD control groups^14^. Detailed information about granular fibrosis stages and stool metabolites was not available in this public dataset. Nevertheless, our validation results were sufficient to confirm the differences in gut-microbiome changes according to fibrosis severity between non-obese and obese subjects. However, external validation using the combined microbiome–metabolite marker in additional Western studies needs to be conducted, considering the possible overfitting error caused by a number of explanatory variables in our study. Fourth, after stratification by obesity and fibrosis stage, each subgroup ended up with a small number of patients, which might lead to an insufficient statistical power prone to a type II error. Finally, we could not identify any clues about clear differences according to fibrosis severity in obese NAFLD, unlike in non-obese NAFLD. This finding might be attributed to the stronger driving force of obesity regarding changes in microbial community structure and their metabolites, such as bile acid composition, interfering with the identification of fibrosis-specific microbial markers in obese subjects with NAFLD.

Despite these limitations, the results from our well-characterized, biopsy-proven NAFLD cohort emphasize not only the importance of the gut microbiota as a risk factor accounting for the pathogenesis of non-obese NAFLD but also the diagnostic and potentially therapeutic implications of the microbiome–metabolite combination as a non-invasive biomarker for diagnosing significant fibrosis in patients with non-obese NAFLD.

## Methods

### Human subjects

We utilized the data from the ‘Boramae NAFLD cohort (NCT 02206841)’ study^5,33^. One hundred seventy-one subjects with biopsy-proven NAFLD and 31 no-NAFLD subjects were included in this study. Subjects with histologically confirmed NAFLD and BMI < 25 kg m^−2^ were classified as non-obese NAFLD subjects. Clinical, metabolic, biochemical, and epidemiological characteristics are described in Supplementary Tables 1–5. This study was performed in accordance with the ethical guidelines of the 1975 Declaration of Helsinki for the participation of human subjects and was approved by the Institutional ‘Review Board of Boramae Medical Center (IRB No. 26-2017-48). Written informed consent was obtained from all of the study subjects.

### Inclusion and exclusion criteria

Briefly, we enrolled eligible subjects from January 2013 to February 2017 prospectively. The inclusion criteria were as follows: (1) adults at least 18 years old; (2) ultrasonographic findings confirming fatty infiltration of the liver; and (3) unexplained elevated ALT levels within the past 6 months. Subjects who met any of the following criteria were excluded: (1) hepatitis B or C virus infection; (2) autoimmune hepatitis, primary biliary cholangitis or primary sclerosing cholangitis; (3) gastrointestinal cancers or hepatocellular carcinoma; (4) drug-induced steatosis or liver injury; (5) Wilson disease or hemochromatosis; (6) excessive alcohol consumption (men: >210 g week^−1^, women: >140 g week^−1^); (7) antibiotics use within the prior month; (8) diagnosis of malignancy (<5 yr); (9) human immunodeficiency virus infection; and (10) chronic disorders associated with lipodystrophy or immunosuppression.

Non‐obese and obese controls included subjects without any suspicion of NAFLD who received liver biopsy: (1) during the evaluation for living donor liver transplantation or (2) during the characterization of solid liver masses that were suspected to be hepatic adenomas or focal nodular hyperplasia based on imaging studies (1 subject in the current cohort with focal nodular hyperplasia confirmed by liver biopsy)^5^. Details of the study subjects and host genotyping are presented in the Supplementary Methods.

### Liver histology

Liver histology was assessed using the NASH CRN histological scoring system by a single liver pathologist. NAFLD was defined as the presence of $\ge$5% macrovesicular steatosis based on histological examination. NASH was determined based on an overall pattern of histological hepatic injury consisting of steatosis, lobular inflammation, and ballooning according to the criteria of Brunt et al.^34,35^ We also scored steatosis, lobular inflammation, and ballooning according to the NAFLD activity scoring system^36^. Fibrosis severity was evaluated according to the criteria of Kleiner et al.^36^.

### Microbiome analysis using 16S rRNA sequencing

DNA in stool samples was extracted using a QIAamp DNA Stool Mini Kit (Qiagen, Hilden, Germany). Sequencing targeting the V4 region of the 16S rRNA was performed using the MiSeq platform (Illumina, San Diego, CA, USA), and further processing of raw sequencing data was performed using the QIIME pipeline (v. 1.8.0)^37^. Details of data processing are presented in the Supplementary Methods.

### Metagenomic shotgun sequencing

DNA in stool samples was extracted using a QIAamp DNA Stool Mini Kit (Qiagen). Sequencing libraries were prepared using an Illumina Nextera DNA Flex Library Prep Kit (Illumina, San Diego, CA, USA). Sequencing was performed on an Illumina NextSeq 500 platform (Illumina). Taxonomic and functional profiles of the microbiome were analyzed using MetaPhlan2 (v. 2.6.0)^38^ and HUMAnN2 (v. 0.11.0)^39^. Details of the analysis are described in the Supplementary Methods.

### Stool metabolite measurements using the GC-FID and Q-TOF system

Stool bile acid profile was assessed using a Q-TOF mass spectrometer (Waters Micromass Technologies, Manchester, UK), and SCFA was measured by using an Agilent Technologies 7890A GC system (Agilent Technologies, Santa Clara, CA, USA) according to David’s method^40^. Details of metabolite analysis are presented in the Supplementary Methods.

### Bioinformatics analysis and statistical tests

For alpha diversity, the OTU table was rarefied to 12,000 sequences per sample, and the Shannon index was measured in QIIME. Nonparametric multi-dimensional scaling (NMDS) plots were depicted using the Vegan package (v. 2.5-6) in R (v.3.5.0)^41^, and the distance was measured using Bray–Curtis methods. Statistical significance between groups was estimated using the adonis function. Multivariate association analysis using microbiome data was performed using MaAsLin (Galaxy v. 1.0)^17^ for the identification of specific taxa associated with the host phenotype without the influence of other metadata. Herein, we adjusted for age, sex, and BMI or clinical factors as fixed variables. Associations in which the *P*-value adjusted by Benjamini and Hochberg’s false discovery rate was <0.25 (default setting) were considered significant. The AUC was measured using SPSS software (v. 25.0) (SPSS Inc., Armonk, NY, USA). For data with non-normal distribution, outliers were removed using the ROUT method^42^ (*Q* = 1%) and statistical comparisons were performed with the Kruskal–Wallis test or the Mann–Whitney test using GraphPad Prism software (v. 8.4.3) (GraphPad Software, San Diego, CA, USA). Details are presented in Supplementary Methods.

### Validation of the data using a Western NAFLD cohort

To validate our results, public dataset reporting microbiome-based biomarkers for diagnosing NAFLD-related cirrhosis was obtained from qiita.microbio.me (QIITA Study 11635)^14^. A total of 168 subjects with metadata on advanced fibrosis and BMI were used for this study. An obese state was defined by the BMI cut-off value (obese, BMI ≥30; non-obese, BMI < 30) currently used in American and European populations^43,44^. Each group was classified into two subgroups according to the presence or absence of advanced fibrosis.

### Interventional animal study

*R. faecis* (JCM no. 15917)^28^ and *V. parvula* (DSM no. 2008) were distributed from the Korean Collection for Type Cultures (KCTC). *R. bromii* was distributed from the American Type Culture Collection (ATCC, no. 27255). *M. funiformis* was isolated from healthy Korean adult feces. Bacteria (10^9^ CFU/mouse) were gavaged orally in C57BL/6 mice with an MCD or a CDAHFD diet. In *db*/*db* mice, *R. faecis* was gavaged orally with a normal chow diet. All of the animal procedures were approved by the Institutional Animal Care and Use Committee of the Seoul National University. The mice were housed in a conventional animal facility according to university guidelines at a light/dark cycle of 12 h, temperature range of 21–24 °C, and humidity range of 40–50%. Details are presented in the Supplementary Methods.

### Reporting summary

Further information on research design is available in the Nature Research Reporting Summary linked to this article.

## Supplementary information

Supplementary Information

Reporting Summary

## Data Availability

The V4 16S rDNA and shotgun metagenome sequence datasets obtained from this study have been deposited in the European Nucleotide Archive databases with the accession codes ERP109777 (https://www.ebi.ac.uk/ena/browser/view/PRJEB27662). All other metadata that support the findings of this study are available from the corresponding author upon reasonable request. A validation cohort was obtained from qiita.microbio.me (QIITA Study 11635, https://www.ebi.ac.uk/ena/browser/view/PRJEB28350). Source data are provided with this paper.

## Source data

Source data

## References

[1] Loomba R, Sanyal AJ (2013). The global NAFLD epidemic. Nat. Rev. Gastroenterol. Hepatol..

[2] Chalasani N (2012). The diagnosis and management of non-alcoholic fatty liver disease: practice guideline by the American Gastroenterological Association, American Association for the Study of Liver Diseases, and American College of Gastroenterology. Gastroenterology.

[3] Leung JC (2017). Histological severity and clinical outcomes of nonalcoholic fatty liver disease in nonobese patients. Hepatology.

[4] Kim D, Kim WR (2017). Nonobese fatty liver disease. Clin. Gastroenterol. Hepatol..

[5] Koo BK (2018). Additive effects of PNPLA3 and TM6SF2 on the histological severity of non‐alcoholic fatty liver disease. J. Gastroenterol. Hepatol..

[6] Begley M, Gahan CG, Hill C (2005). The interaction between bacteria and bile. FEMS Microbiol. Rev..

[7] Cani PD (2007). Metabolic endotoxemia initiates obesity and insulin resistance. Diabetes.

[8] Dawes E, Foster SM (1956). The formation of ethanol in *Escherichia coli*. Biochim. Biophys. Acta.

[9] Zhu L (2013). Characterization of gut microbiomes in nonalcoholic steatohepatitis (NASH) patients: a connection between endogenous alcohol and NASH. Hepatology.

[10] Wang Z (2011). Gut flora metabolism of phosphatidylcholine promotes cardiovascular disease. Nature.

[11] Loomba R (2017). Gut microbiome-based metagenomic signature for non-invasive detection of advanced fibrosis in human nonalcoholic fatty liver disease. Cell Metab..

[12] Boursier J (2016). The severity of nonalcoholic fatty liver disease is associated with gut dysbiosis and shift in the metabolic function of the gut microbiota. Hepatology.

[13] Bajaj JS (2014). Altered profile of human gut microbiome is associated with cirrhosis and its complications. J. Hepatol..

[14] Caussy C (2019). A gut microbiome signature for cirrhosis due to nonalcoholic fatty liver disease. Nat. Commun..

[15] Qin N (2014). Alterations of the human gut microbiome in liver cirrhosis. Nature.

[16] He Y (2018). Regional variation limits applications of healthy gut microbiome reference ranges and disease models. Nat. Med..

[17] Morgan XC (2012). Dysfunction of the intestinal microbiome in inflammatory bowel disease and treatment. Genome Biol..

[18] Qin J (2012). A metagenome-wide association study of gut microbiota in type 2 diabetes. Nature.

[19] Lim MY (2016). The effect of heritability and host genetics on the gut microbiota and metabolic syndrome. Gut.

[20] Wu H (2017). Metformin alters the gut microbiome of individuals with treatment-naive type 2 diabetes, contributing to the therapeutic effects of the drug. Nat. Med..

[21] Marchandin, H. & Jumas-Bilak, E. In *The Prokaryotes* (Springer, 2014).

[22] Kara D, Luppens SB, ten Cate JM (2006). Differences between single‐and dual‐species biofilms of *Streptococcus mutans* and *Veillonella parvula* in growth, acidogenicity and susceptibility to chlorhexidine. Eur. J. Oral. Sci..

[23] Kummen M (2016). The gut microbial profile in patients with primary sclerosing cholangitis is distinct from patients with ulcerative colitis without biliary disease and healthy controls. Gut.

[24] Chen Y (2011). Characterization of fecal microbial communities in patients with liver cirrhosis. Hepatology.

[25] Duarte SMB (2017). Gut microbiome composition in lean patients with NASH is associated with liver damage independent from caloric intake: a prospective pilot study. Nutr. Metab. Cardiovasc. Dis..

[26] Wang B (2016). Altered fecal microbiota correlates with liver biochemistry in nonobese patients with non-alcoholic fatty liver disease. Sci. Rep..

[27] Munukka E (2017). *Faecalibacterium prausnitzii* treatment improves hepatic health and reduces adipose tissue inflammation in high-fat fed mice. ISME J..

[28] Kim MS, Roh SW, Bae JW (2011). *Ruminococcus faecis* sp. nov., isolated from human faeces. J. Microbiol..

[29] Jiao N (2018). Suppressed hepatic bile acid signalling despite elevated production of primary and secondary bile acids in NAFLD. Gut.

[30] Tian Y (2020). The microbiome modulating activity of bile acids. Gut Microbes.

[31] Sinha SR (2020). Dysbiosis-induced secondary bile acid deficiency promotes intestinal inflammation. Cell Host Microbe.

[32] Anstee QM, Targher G, Day CP (2013). Progression of NAFLD to diabetes mellitus, cardiovascular disease or cirrhosis. Nat. Rev. Gastroenterol. Hepatol..

[33] Koo BK (2017). Sarcopenia is an independent risk factor for non-alcoholic steatohepatitis and significant fibrosis. J. Hepatol..

[34] Brunt EM, Janney CG, Di Bisceglie AM, Neuschwander-Tetri BA, Bacon BR (1999). Nonalcoholic steatohepatitis: a proposal for grading and staging the histological lesions. Am. J. Gastroenterol..

[35] Brunt EM, Kleiner DE, Wilson LA, Belt P, Neuschwander‐Tetri BA (2011). Nonalcoholic fatty liver disease (NAFLD) activity score and the histopathologic diagnosis in NAFLD: distinct clinicopathologic meanings. Hepatology.

[36] Kleiner DE (2005). Design and validation of a histological scoring system for nonalcoholic fatty liver disease. Hepatology.

[37] Caporaso JG (2010). QIIME allows analysis of high-throughput community sequencing data. Nat. Methods.

[38] Truong DT (2015). MetaPhlAn2 for enhanced metagenomic taxonomic profiling. Nat. Methods.

[39] Franzosa EA (2018). Species-level functional profiling of metagenomes and metatranscriptomes. Nat. Methods.

[40] David LA (2014). Diet rapidly and reproducibly alters the human gut microbiome. Nature.

[41] Oksanen, J. et al. The vegan package. *Community Ecology Package* **10**, 719 (2007).

[42] Motulsky HJ, Brown RE (2006). Detecting outliers when fitting data with nonlinear regression–a new method based on robust nonlinear regression and the false discovery rate. BMC Bioinforma..

[43] Organization, W. H. Physical status: the use and interpretation of anthropometry. Report of a WHO Expert Consultation. *WHO Technical Report Series* (1995). 8594834

[44] Hedley AA (2004). Prevalence of overweight and obesity among US children, adolescents, and adults, 1999–2002. JAMA.

