# Supplementary material for: Distinct signatures of gut microbiome and metabolites associated with significant fibrosis in non-obese NAFLD
Source: Nat Commun. 2020 Oct 5;11:4982. doi: 10.1038/s41467-020-18754-5 (PMC7536225; doi:10.1038/s41467-020-18754-5)
Supplement: Supplementary file 1 — Supplementary Information [file 41467_2020_18754_MOESM1_ESM.pdf]

Supplemental information

Distinct signatures of gut microbiome and metabolites associated with significant fibrosis in biopsy-proven non-obese NAFLD

Lee et al.

Supplementary Methods

Human subject

We utilized the data from the 'Boramae NAFLD cohort (NCT 02206841)' study. The homeostasis model assessment of insulin resistance (HOMA-IR) and adipose tissue insulin resistance (adipo-IR) were calculated as described elsewhere[1,2]. The fibrosis 4 index (FIB-4), a non-invasive fibrosis marker, was calculated using the appropriate equation[3]. We obtained two different liver tissue samples at the time of liver biopsy for a solid liver mass: one sample from the hepatic lesion and the other from the surrounding normal liver parenchyma. Liver biopsy performed in these controls showed <5% macrovesicular steatosis with no histological evidence of NASH[4]. Among the eligible subjects, liver biopsy was performed if at least two of the following risk factors were present: high triglycerides levels; low high-density lipoprotein (HDL)-cholesterol level; abdominal obesity; hypertension; the presence of diabetes mellitus and/or insulin resistance; or clinically suspected NASH or hepatic fibrosis. This study was performed in accordance with the ethical guidelines of the 1975 Declaration of Helsinki for the participation of human subjects and was approved by the Institutional `Review Board of Boramae Medical Center (IRB No. 26-2017-48). Written informed consent was obtained from all of the study subjects.

Host genotyping

Established risk alleles for NAFLD were selected for genotyping as described elsewhere[5]. Specifically, the *PNPLA3* rs738409 C>G (I148M)[6-8], *TM6SF2* rs58542926 C>T (E167K)[9,10], *MBOAT7-TMC4* C>T rs641738[11,12], and *SREBF-2* rs133291 C>T[13] single-nucleotide polymorphisms were genotyped in the entire cohort by using TaqMan 50-nuclease assays (Life Technologies, Carlsbad, CA, USA) according to the manufacturer's instructions. Hardy–Weinberg equilibrium was confirmed using the chi-square test.

Microbiome analysis using 16S rRNA sequencing

Subjects' stool samples were immediately frozen at −80°C and transferred to the laboratory for microbiome analysis. Two hundred-milligram aliquots of stool samples were used for analysis, and DNA in stool samples was extracted using a QIAamp DNA

Stool Mini Kit (Qiagen, Hilden, Germany). Sequencing was performed using the MiSeq platform (Illumina, San Diego, CA), and further processing of raw sequencing data was performed using the QIIME pipeline (v. 1.8.0). To improve the DNA extraction efficiency, an additional bead-beating step was included. After preprocessing the samples, the remaining steps were performed using a QIAcube (Qiagen) instrument to maintain consistent efficiency. PCR amplification of the extracted DNA was performed with the Illumina-adapted universal primers 515F/806R targeting the V4 region of the 16S rRNA gene[14], and the amplicon was purified using the QIAquick PCR Purification Kit (Qiagen). Quantification of the amplicon was performed using the KAPA Library Quantification Kit (KAPA Biosystems, Boston, MA, USA). The samples were then pooled and used as input for the MiSeq platform (Illumina, San Diego, CA, USA). A total of 7,276,554 sequencing reads were generated from 202 samples for an average of 36,022 reads per sample. Sequencing data were processed using the QIIME pipeline (v. 1.8.0)[15]. Operational taxonomic unit (OTU) picking and assigning sequences to an OTU were performed at the 97% similarity level against the gg_13_5 Greengenes database[16]. Representative sequences were chosen using UCLUST software and aligned through the PyNAST algorithm (v. 1.2.2)[17]. The ribosomal database project classifier[18] was used to assign taxa to OTUs, and chimeric sequences were removed using the ChimeraSlayer algorithm (v. microbiomutil-r20110519)[19]. The relative abundance table from the phylum to genus level was used for further microbiome analysis.

Metagenomic shotgun sequencing

DNA in stool samples was extracted using a QIAamp DNA Stool Mini Kit (Qiagen, Hilden, Germany) and quantified using a Quant-iT™ PicoGreen™ dsDNA Assay kit (Thermo Fisher Scientific, Waltham, MA, USA). Integrity of total DNA was checked using gel electrophoresis. Sequencing libraries were prepared using an Illumina Nextera DNA Flex Library Prep Kit (Illumina, San Diego, CA, USA) and was checked using a Caliper LabChip GX analyzer (PerkinElmer, Waltham, MA, USA). Sequencing was performed on an Illumina NextSeq 500 platform (Illumina) to produce 75 bp paired-end sequences. Metagenomes averaged 8,566,242 ± 1,378,228 reads (mean ± s.d.) per sample before filtering and 8,131,721 ± 1,314,620 reads per sample

afterward. To remove low-quality reads and human contamination, sequence reads were processed with KneadData (v. 0.6.1), which uses the Trimmomatic (v. 0.33)[20] and Bowtie2 (v. 2.3.2)[21]. Taxonomic profiling of the metagenome was performed with MetaPhlAn2 (v. 2.6.0)[22] and functional profiling was performed with HUMAnN2 (v. 0.11.1)[23] using the UniRef50[24] database.

Co-occurring and co-excluding interactions in the microbial ecological network

For network analysis, correlations were measured using the SparCC (v. 0.1.0) package in R (v. 3.5.0)[25] and were visualized using Cytoscape (v. 3.7.1)[26]. In the current study, the raw OTU table was processed in 3 steps: 1. rarefaction with 12,000 sequences; 2. collapsing to the family level; and 3. identifying OTUs present in more than 15% of all of the subjects. Pseudo *P*-values were calculated with 500 bootstraps in the R package, and *P*-values (*q*-value) adjusted by Benjamini and Hochberg's FDR less than 0.25 were included.

Bile acid measurements using a UPLC/Q-TOF system

Two hundred-milligram aliquots of stool samples were thawed, and 4 volumes of distilled water were added followed by vortexing at room temperature for 5 min until the samples were homogenized. After centrifugation at 14,000*g* for 5 min, the supernatants were transferred to new tubes for further analysis. One volume of 80% methanol (Merk Millipore, Billerica, MA, USA) was added to the supernatant, followed by vortexing for 1 min. Samples were sonicated for 3 min and centrifuged at 14,000*g* for 2 min. The supernatants were transferred to new tubes. For second extraction, 1 mL of 100% methanol was added to the precipitate. After extraction using a homogenizer for 30 min with 15 Hz, the remaining solid material was removed by centrifugation (14,000 *g* for 3 min). Supernatant was added to the existing tube. The combined supernatant was evaporated using a vacuum concentrator (Eppendorf, Hamburg, Germany) for more than 9 hours. Finally, samples were resuspended in 500 mL 55%/45% methanol/water (v/v) and then filtered using a 0.22-µm filter (Merck Millipore). Mouse bile acid measurements from cecum samples were identical to those from humans except that 10 volumes of 80%

MeOH were added to the cecum sample for the first extraction. Serially diluted mixtures of bile acids were used as a standard.

Cholic acid (CA), chenodeoxycholic acid (CDCA), deoxycholic acid (DCA), lithocholic acid (LCA), ursodeoxycholic acid (UDCA), taurocholic acid (TCA), taurolithocholic acid (TLCA), taurodeoxycholic acid (TDCA), tauroursodeoxycholic acid (TUDCA), glycochenodeoxycholic acid (GCDCA), glycodeoxycholic acid (GDCA), and glycoursodeoxycholic acid (GUDCA) were purchased from Sigma-Aldrich (St.Louis, MO, USA). Glycocholic acid (GCA) was purchased from Calbiochem (San Diego, CA, USA). α-muricholic acid (α-MCA), β-muricholic Acid (β-MCA), ω-muricholic acid (ω-MCA), tauro-α-muricholic acid (Tα-MCA), and tauro-β-muricholic acid (Tβ-MCA) were purchased from Cayman Chemical (Ann Arbor, MI, USA). After the separation of bile acids according to Buffie's method[27], profiling of stool bile acids was performed using a Q-TOF mass spectrometer (Waters Micromass Technologies, Manchester, UK). Quantification of bile acids was performed using QuanLynx™ software (v. 4.1) and MassLynx™ (v. 4.1) (Waters Corp., Milford, MA, USA).

Stool short-chain fatty acid (SCFA) measurements using the GC-FID system

Supernatants described in bile acid measurements were also used for SCFA measurements. For fecal SCFA measurements, 5% of 95% sulfuric acid (Sigma-Aldrich) was added to the supernatants for acidification, followed by vortexing for 5 min at room temperature. To extract volatile materials, 10% internal standard (1% 2-methyl pentanoic acid) (Sigma-Aldrich) and 1 volume of anhydrous ethyl ether (Sigma-Aldrich) were added. The samples were vortexed for 1 min and then centrifuged at 14,000*g* for 5 min. The upper layer was carefully moved to a new tube, and a mixture containing 10 mM acetate (Sigma-Aldrich), butyrate (Sigma-Aldrich), and propionate (Sigma-Aldrich) was used as the standard for SCFA analysis. For mouse SCFAs measurements from cecum samples, 4 volumes of distilled water were added to samples, followed by vortexing for 5 min. Five percent of 95% sulfuric acid was added to the samples for acidification, followed by stabilization for 5 min. After centrifugation at 14,000*g* for 5 min, the supernatants were transferred to a new tube. Other steps were identical to human stool SCFAs measurements. SCFAs were

measured by using an Agilent Technologies 7890A GC system (Agilent Technologies, Santa Clara, CA, USA) according to David's method[28].

Prediction of significant fibrosis by the ROC curve analysis

To validate the ability of microbiome-based biomarkers to predict significant fibrosis, the area under the receiver operating characteristic curve (AUC) method was used. The relative abundances of two family-level bacteria identified in the current study, four metabolites (CA, CDCA, UDCA, and propionate), and FIB-4 were used as inputs for the AUC, and the combination of these factors was calculated using binary logistic regression in SPSS software (v. 25.0) (SPSS Inc., Armonk, NY, USA). The AUROC comparison was performed by the DeLong test using MedCalc software (v. 18.2.1) (MedCalc Software BVBA, Mariakerke, Belgium).

Mouse intervention study

Six-week-old of male C57BL/6N mice were purchased from Orient Bio (Seongnam, Republic of Korea), and 5 week-old male C57BLKS/J-db/db and control C57BLKS/J-m+/db mice were purchased from SLC (Shizuoka, Japan). The mice were housed in a conventional animal facility according to university guidelines. All of the animal procedures were approved by the Institutional Animal Care and Use Committee. After 1 weeks of acclimatization on a standard chow diet, the mice were treated with streptomycin (1g/L) in drinking water for the colonization of administered bacteria. For the subsequent 5 weeks, the mice were fed the methionine-and choline-deficient, L-amino acid diet (MCD) (Research Diet, New Brunswick, NJ, USA; Cat. no.: A02082002B). *db/db* mice were fed a normal chow diet.
For the subsequent 8 weeks, the mice were fed the choline-deficient, L-amino acid-defined, high-fat diet (CDAHFD) (Research Diet, Cat. no.: A06071302). The mice were gavaged daily with 200 μL of either bacteria (10^9 CFU/mouse in PBS) or sham. An intraperitoneal glucose tolerance test (ipGTT) of *db/db* mice was performed after three weeks of bacteria challenge. The mice were fasted for 16 h with free access to water and a solution of glucose (1 g glucose/kg body weight) was administered by intraperitoneal injection. Blood glucose was measured at different time points in tail vein blood using a glucometer (Accu-Chek Performa, Roche Diagnostic, Mannheim,

Germany). Serum ALT and AST levels were measured using a chemical analyzer (Fuji DRI-CHEM 3500i, Fuji Photo Film, Tokyo, Japan) or commercial kits (EnzyChrom Alanine Transaminase Assay kit, BioAssay Systems, Hayward, CA, USA; EnzyChrom Aspartate Transaminase Assay kit, BioAssay Systems). Serum insulin levels were measured using an Ultra Sensitive Mouse Insulin ELISA kit (Crystal Chem, Downers Grove, IL, USA). For quantitative PCR, total RNA from liver samples was extracted using the easy-spin Total RNA Extraction Kit (iNtRON Biotechnology, Seongnam, Republic of Korea) and reverse transcribed to cDNA using a High Capacity RNA-to-cDNA kit (Thermo Fisher Scientific, Waltham, MA, USA). Quantitative PCR was performed using the SYBR Green qPCR Master mix (Thermo Fisher Scientific) and the Quantstudio 6 Flex qPCR System. The primer sequences were as follows: *Cyclophilin A* forward: 5'-TGGAGAGCACCAAGACAGACA-3', reverse: 5'-TGCCGGAGTCGACAATGAT-3'; *Col1a1* forward: 5'-ACCTGTGTGTTCCCTACTCA-3', reverse: 5'-GACTGTTGCCTTCGCCTCTG-3'; *Timp1* forward: 5'-GGTGTGCACAGTGTTTCCCTGTTT-3', reverse: 5'-TCCGTCCACAAACAGTGAGTGTCA- 3'; and *α-SMA* forward: 5'-GGCTCTGGGCTCTGTAAGG-3', reverse: 5'-CTCTTGCTCTGGGCTTCATC-3'.

Cultivation of bacteria

R. faecis and *V. parvula* were distributed from KCTC (KCTC nos. 5757 and 5019). *R. bromii* was distributed from ATCC (ATCC no. 27255). *M. funiformis* was isolated from healthy Korean adult feces. Bacteria were cultivated under anaerobic conditions (Coy Laboratory Products Inc., Grass Lake, MI, USA). *R. faecis* and *M. funiformis* were cultivated in YBHI medium[29]. *V. parvula* was cultivated in GAM medium (MBcell, Seoul, Korea) supplemented with sodium lactate solution (15g/1L, Sigma-Aldrich) and putrescine (3mg/1L, Sigma-Aldrich). *M. funiformis* was cultivated in YCFAG medium[30]. Bacterial cells were harvested by centrifugation and washed using 1x PBS (+0.5% cysteine). After repeated washing, bacterial cells were resuspended using 1x PBS for oral gavage (final concentration (10^9 CFU/mouse).

Mouse histological analysis

After euthanasia, live samples were excised and fixed in 10% formalin solution (Sigma-Aldrich). Hematoxylin and eosin (H&E) and Sirius red staining were performed at a core facility (LOGONE Bio Convergence Research Foundation, Seoul, Repulic of Korea). Stained whole-slide images were analyzed using the Pannoramic Viewer (3DHISTECH, Budapest, Hungary). For calculating collagen proportionate area (CPA), eight images per group were randomly chosen and analyzed using ImageJ software (NIH, Bethesda, MD, USA; http://imagej.nih.gov/ij).

Statistical analysis

Statistical significance between two groups was calculated with the two-sided Mann-Whitney test. Among three groups, the Kruskal-Wallis test with Dunn's multiple comparisons test was performed using GraphPad Prism software Ver. 7.0d (GraphPad Software, San Diego, CA, USA).

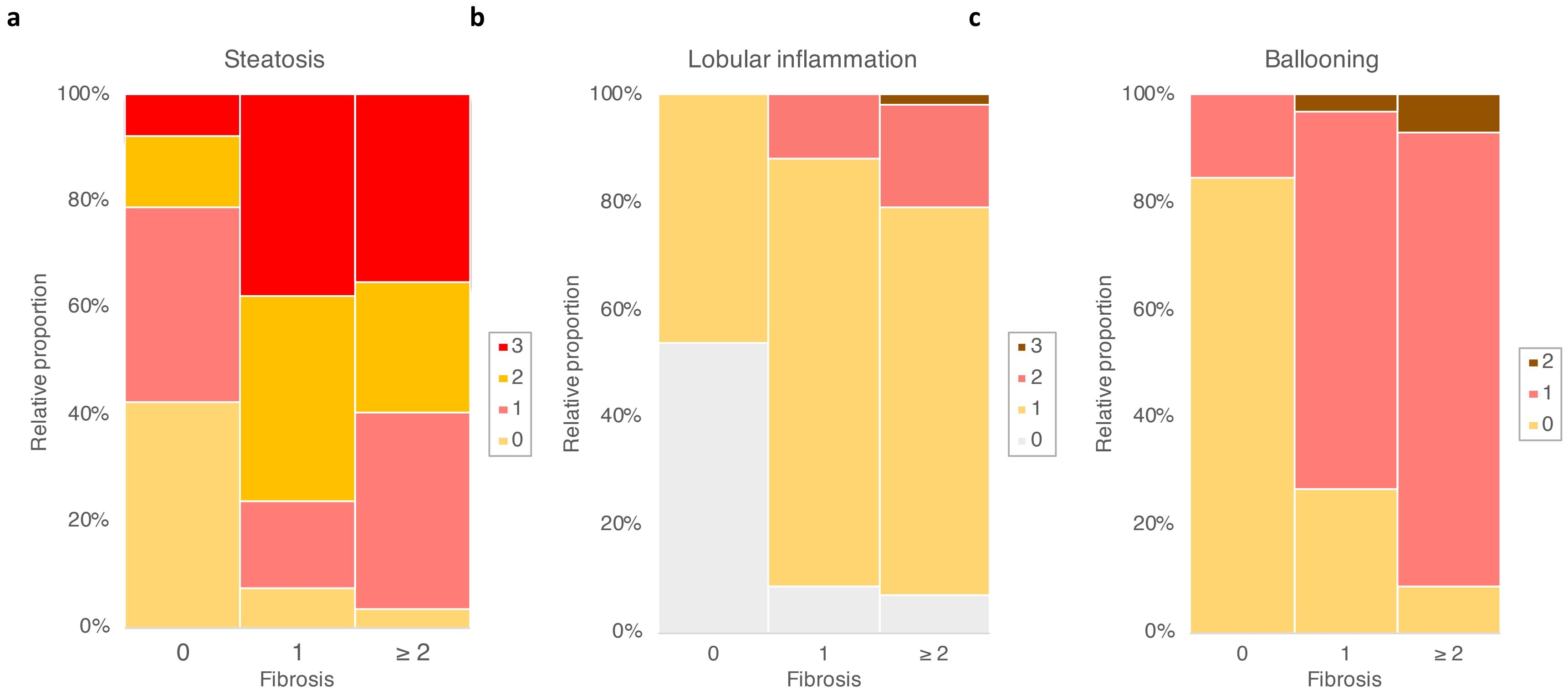

Supplementary Figure 1 Histological distribution of study subjects stratified by fibrosis severity. Relative proportions of histologic features, including (a) steatosis, (b) lobular inflammation, and (c) ballooning, are depicted as stacked bar plots (*n*=52 (F0), 93 (F1), 57 (F≥2)).

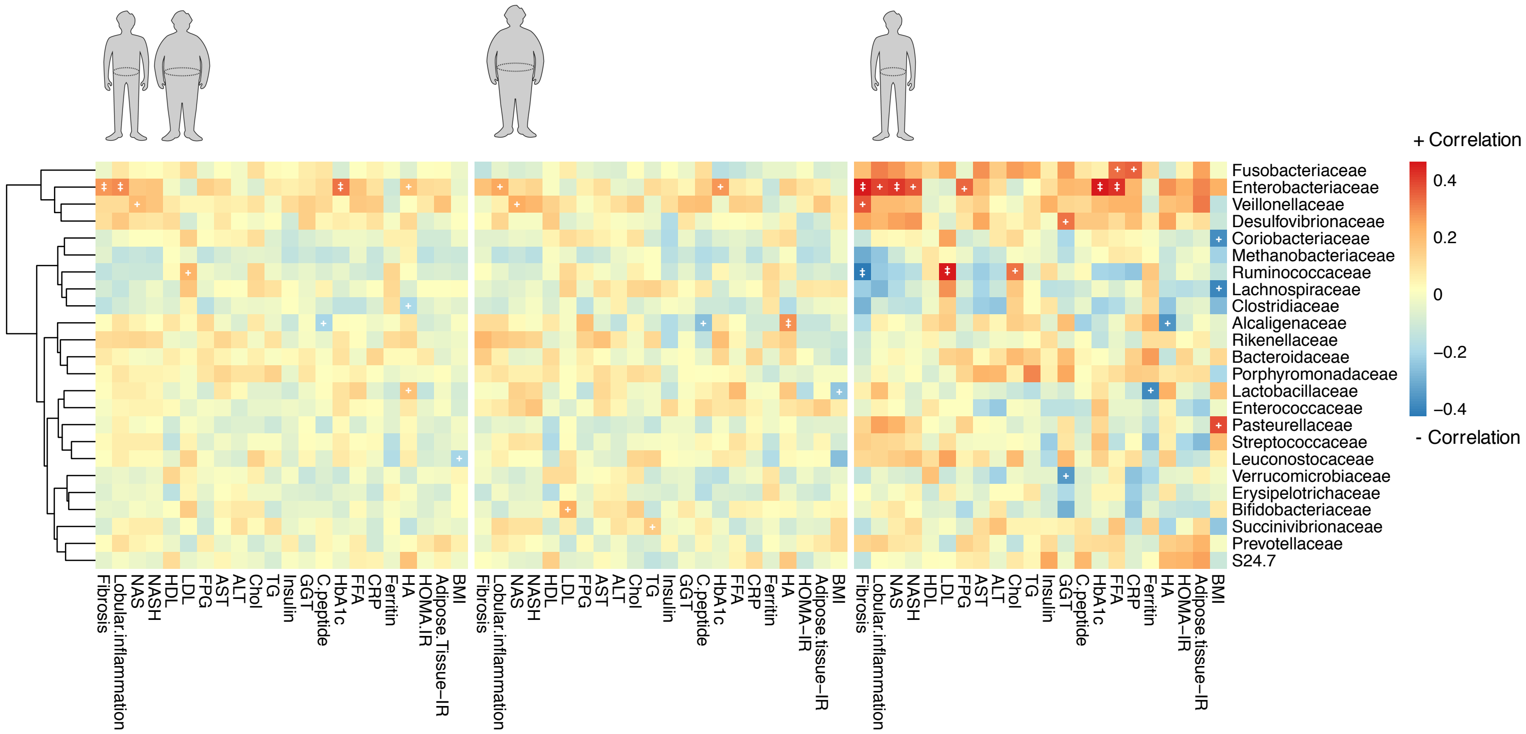

Supplementary Figure 2 Correlations between microbial taxa and host metabolic markers in all, obese, and non-obese subjects. The correlation coefficients between the relative abundances of 24 family taxa and 22 clinical metadata variables were calculated using a Spearman's nonparametric correlation and visualized using heatmap analysis. The color of the matrix denotes the degree of correlation between the taxa and metadata variables (all, *n*=202; obese, *n*=138; non-obese, *n*=64). +*P*<0.05, ‡*P*<0.001. Abbreviations: BMI, body mass index; Adipose tissue-IR, adipose tissue insulin resistance; HOMA-IR, homeostasis model assessment of insulin resistance; HA, hyaluronic acid; hsCRP, high sensitivity C-reactive protein; FFA, free fatty acid; HbA1c, glycosylated hemoglobin; GGT, gamma-glutamyl transferase; TG, triglycerides; Chol, cholesterol; ALT, alanine transaminase; AST, aspartate transaminase; FPG, fasting plasma glucose; LDL, low-density lipoprotein; HDL, high-density lipoprotein; NAS, nonalcoholic fatty liver disease activity score.

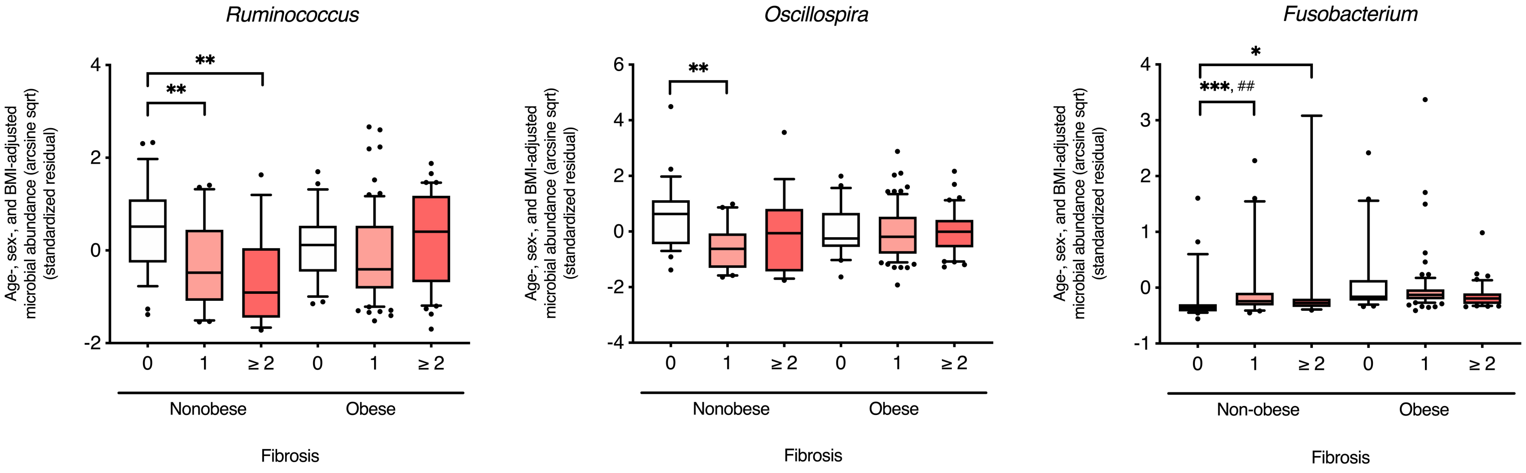

Supplementary Figure 3 Associations between the relative abundances of specific gut microbial taxa at the genus level and fibrosis severity stratified by obesity status. Arcsine-root transformed abundances of taxa were regressed against age, sex, and BMI according to fibrosis severity, and the standard residuals are expressed as box plots. The box plots indicate the median, 25th to 75th percentiles (boxes), and 10th to 90th percentiles (whiskers) [from left, *Ruminococcus*, *P*=0.0098, 0.0011; *Oscillospira*, *P*=0.0027; *Fusobacterium*, *P*=0.0002, *q*=0.0449, *P*=0.0458; non-obese, *n*=27 (F0), 20 (F1), 17 (F≥2); obese, *n*=25 (F0), 73 (F1), 40 (F≥2)]. Statistical analyses for multivariate associations were performed using the MaAsLin pipeline with false discovery rate (q value). *P<0.05, **P<0.01, ***P<0.001, ##q<0.05

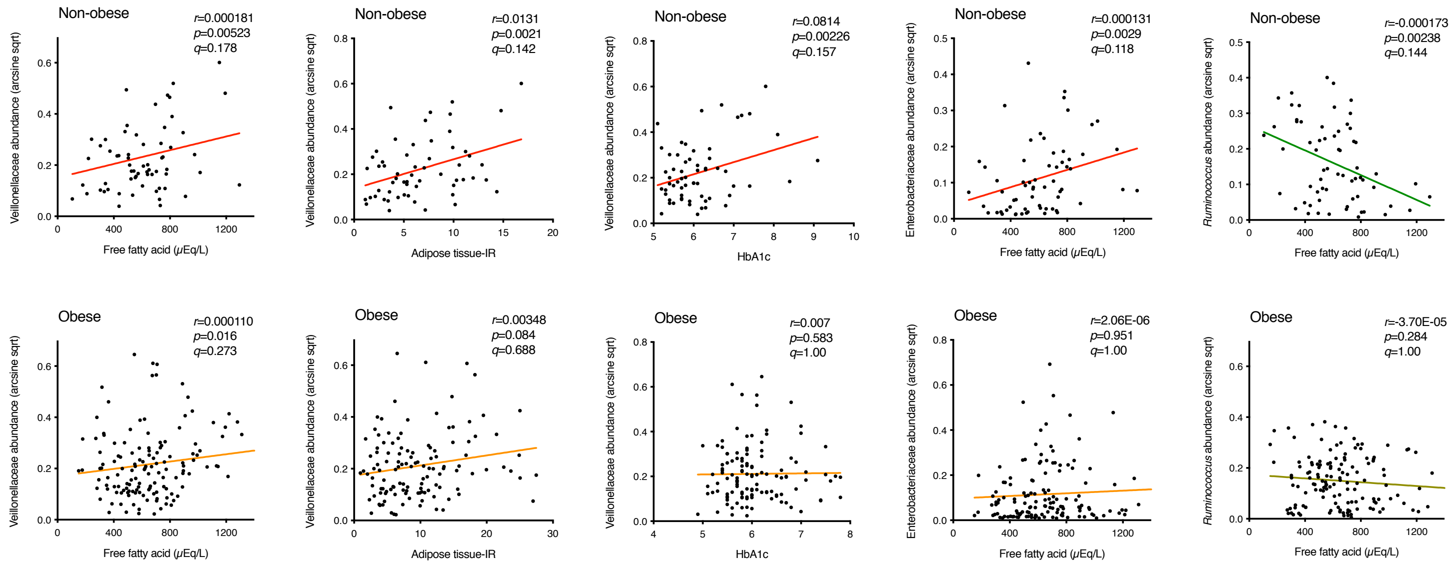

Supplementary Figure 4 Associations between the relative abundances of specific gut microbiota and host metabolic markers in non-obese and obese subjects. Associations between metabolic traits and the arcsine-root transformed abundances of bacteria were measured by multivariate association with linear models (MaAsLin) with adjustments for multiple comparisons (q value). Significant associations regressed against sex, age, and BMI are depicted. Lines represent the linear model fit. *r*, correlation coefficient (non-obese, *n*=64; obese, *n*=138). Abbreviations: Adipose tissue-IR, adipose tissue insulin resistance; HbA1c, glycosylated hemoglobin.

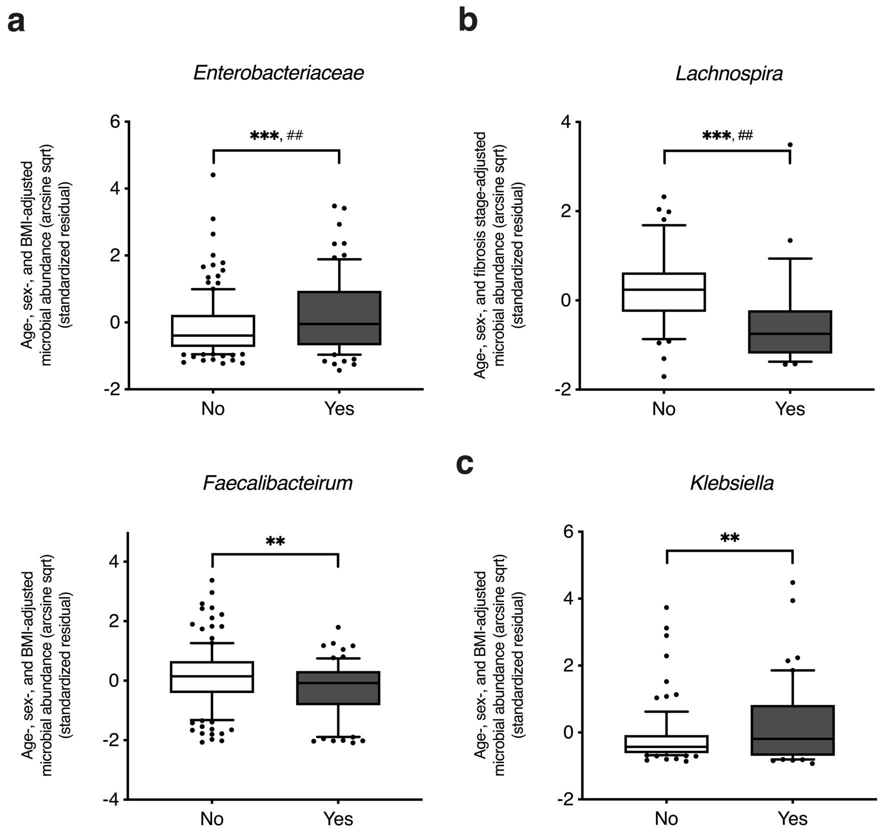

Supplementary Figure 5 Associations between the relative abundances of specific gut microbiome components and the presence or absence of diabetes mellitus. Arcsine-root transformed abundances of taxa were regressed against age, sex, and BMI according to diabetes mellitus, and the standard residuals are expressed as box plots (a, all; b, non-obese subjects; c, obese subjects). The box plots indicate the median, 25th to 75th percentiles (boxes), and 10th to 90th percentiles (whiskers) [(a)(top) *P*=0.0002, q=0.0390, (bottom) *P*=0.0029; (b) *P*>0.001, q=0.0316; (c) *P*=0.0026], [(yes) *n*=72, (no) *n*=130)]. Statistical analyses for multivariate associations were performed using the MaAsLin pipeline with false discovery rate (q value). **P*<0.05, ***P*<0.01, ****P*<0.001, $^{##}$q<0.05. Source data are provided as a Source Data file.

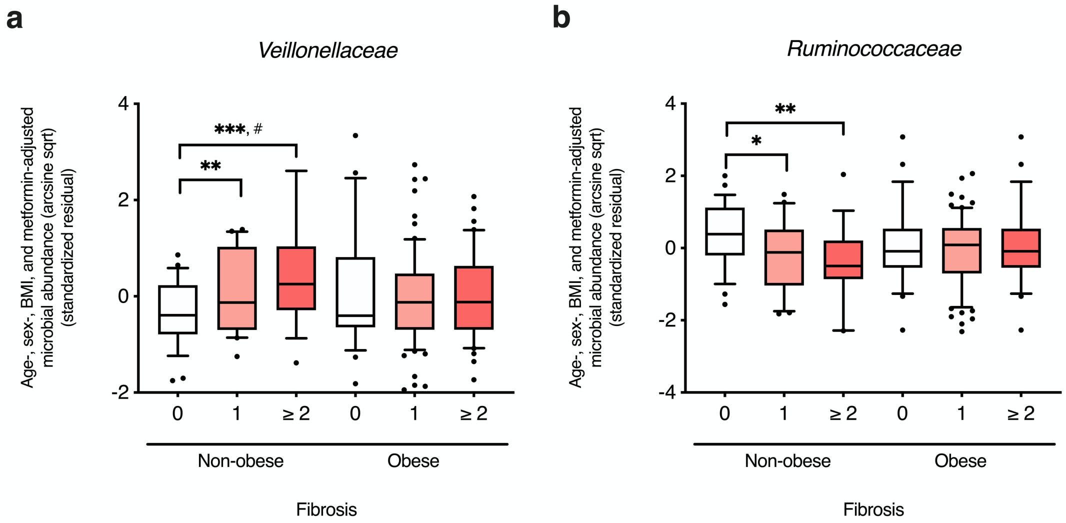

Supplementary Figure 6 Associations between the relative abundances of specific gut microbiome components and the metformin use. Arcsine-root transformed abundances of taxa were regressed against age, sex, BMI, and the use of metformin according to fibrosis severity, and the standard residuals are expressed as box plots (a, *Veillonellaceae*; b, *Ruminococcaceae*). The box plots indicate the median and 25th to 75th percentiles (boxes) and 10th to 90th percentiles (whiskers). Statistical analyses for multivariate associations were performed using the MaAsLin pipeline with false discovery rate (q value) [from left (a) *P*=0.0056, 0.0002, *q*=0.0602; (b) *P*=0.0313, 0.0012], [non-obese, *n*=27 (F=0), 20 (F=1), 17 (F≥2); obese, *n*=25 (F=0), 73 (F=1), 40 (F≥2)]. **P*<0.05, ***P*<0.01, ****P*<0.001, #q<0.10. Source data are provided as a Source Data file.

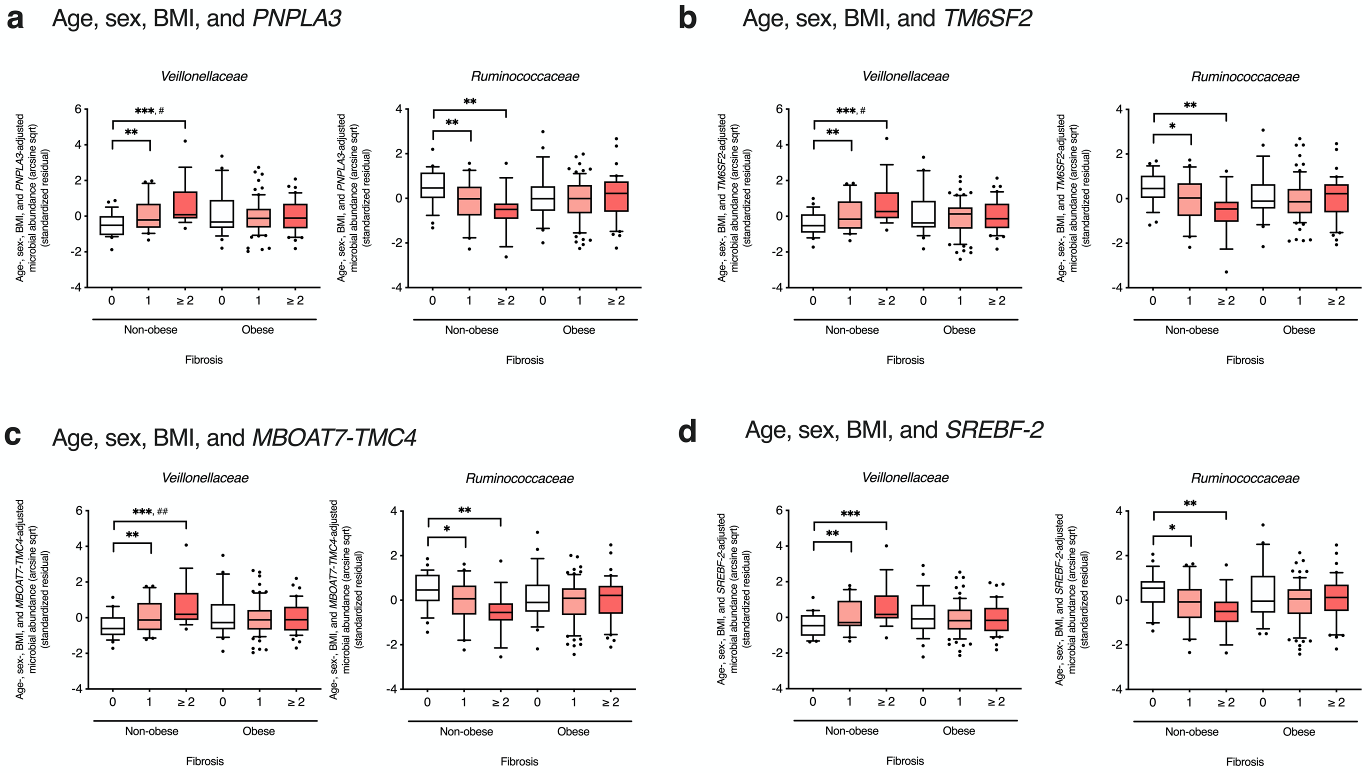

Supplementary Figure 7 Multivariate associations between specific gut microbiome components and fibrosis severity adjusted for host genetic factors. Arcsine-root transformed abundances of bacteria were regressed against age, sex, BMI, and (a) *PNPLA3*, (from left *P*=0.0052, 0.0005, *q*=0.0805, *P*=0.0321, 0.0018) (b) *TM6SF2* (from left, *P*=0.0056, 0.0002, *q*=0.0602, *P*=0.0313, 0.0056) (c) *MBOAT7-TMC4* (from left, *P*=0.0056, 0.0002, *q*=0.0488, *P*=0.0313, 0.0056), and (d) *SREBF-2* (from left, *P*=0.0067, 0.0006, 0.0271, 0.0023). Statistical significance was calculated using multivariate association with linear models (MaAsLin) with adjustments for multiple comparisons (q value). The box plots indicate the median and 25th to 75th percentiles (boxes) and 10th to 90th percentiles (whiskers)

[non-obese, *n*=27 (F=0), 20 (F=1), 17 (F≥2); obese, *n*=25 (F=0), 73 (F=1), 40 (F≥2)]. **P*<0.05, ***P*<0.01, ****P*<0.001, $^{\#}$q<0.10, $^{\#\#}$q<0.05. Source data are provided as a Source Data file.

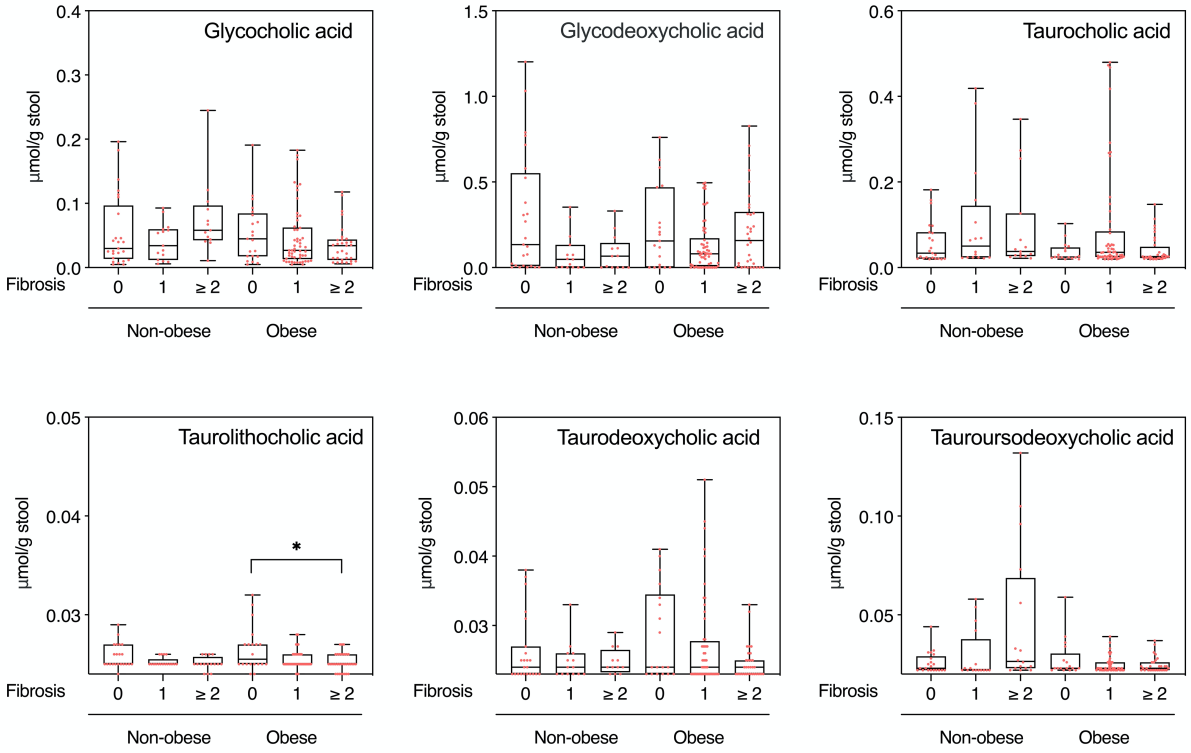

Supplementary Figure 8 Comparison of stool conjugated bile acids levels stratified by fibrosis severity and obesity status. The box plots represent the relative abundance of stool bile acid levels, which are stratified by fibrosis severity and obesity status. The box plots indicate the median and 25th to 75th percentiles (boxes) and minimum to maximum (whiskers) (taurolithocholic acid, *P*=0.0479) (non-obese, *n*=27 (F0), 20 (F1), 17 (F≥2); obese, *n*=25 (F0), 73 (F1), 40 (F≥2)). Outliers were removed by the ROUT method (Q=1%) and data were analyzed using the nonparametric Kruskal-Wallis test with Dunn's multiple comparison test. **P*<0.05, ***P*<0.01.

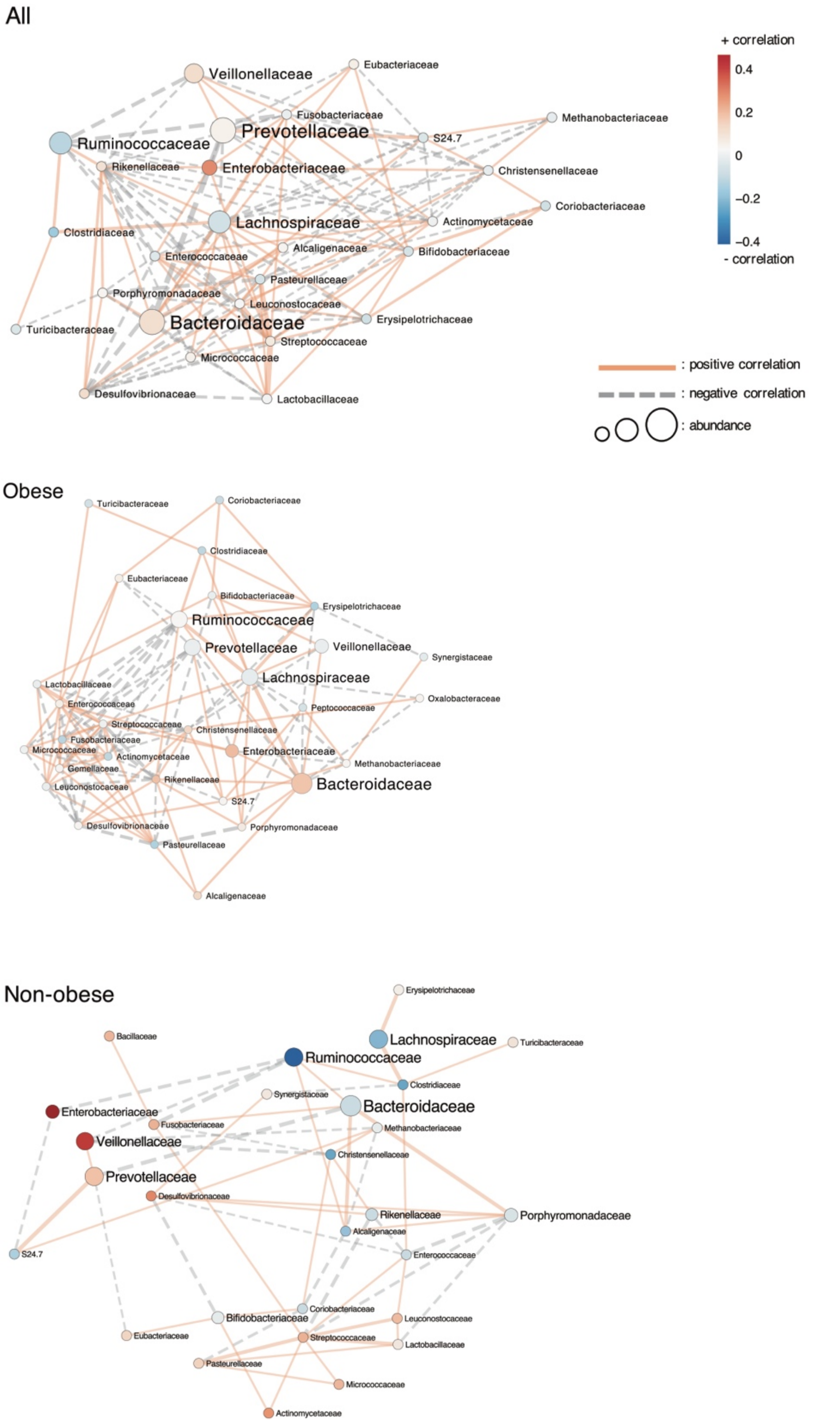

Supplementary Figure 9 Co-occurrence analysis of the specific gut microbiota in all, obese, and non-obese subjects

Co-occurrence coefficients among family-level microbiota were calculated by SparCC and only significant networks (*P*<0.05) are depicted using Cytoscape. The solid line (orange) and dotted line (gray) indicate positive and negative correlations, respectively. The size of the node denotes the relative abundance of the bacteria, and the color indicates the degree of correlation with fibrosis severity. The *P*-value for each

coefficient was obtained by bootstrapping the data set 500 times and applying SparCC to each of those 500 data sets.

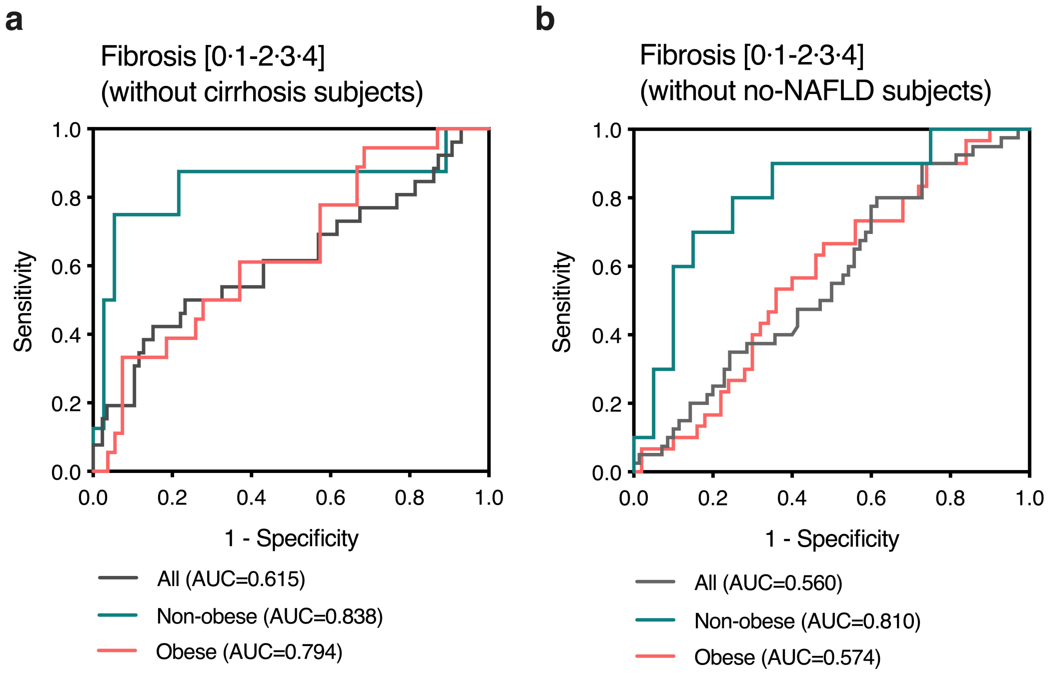

Supplementary Figure 10 Receiver-operating characteristic (ROC) curves for the diagnosis of significant fibrosis after excluding cirrhotic or no-NAFLD subjects.
ROC curves using the combination of two bacteria (*Veillonellaceae* and *Ruminococcaceae*) and four stool metabolites (CA, CDCA, UDCA, and propionate) were plotted for the diagnosis of significant fibrosis and the areas under the ROC curves (AUCs) were calculated. (a) Cirrhotic subjects were excluded for diagnosing significant fibrosis in all, non-obese, and obese subjects. (b) No-NAFLD subjects were excluded for diagnosing significant fibrosis in all, non-obese, and obese subjects.

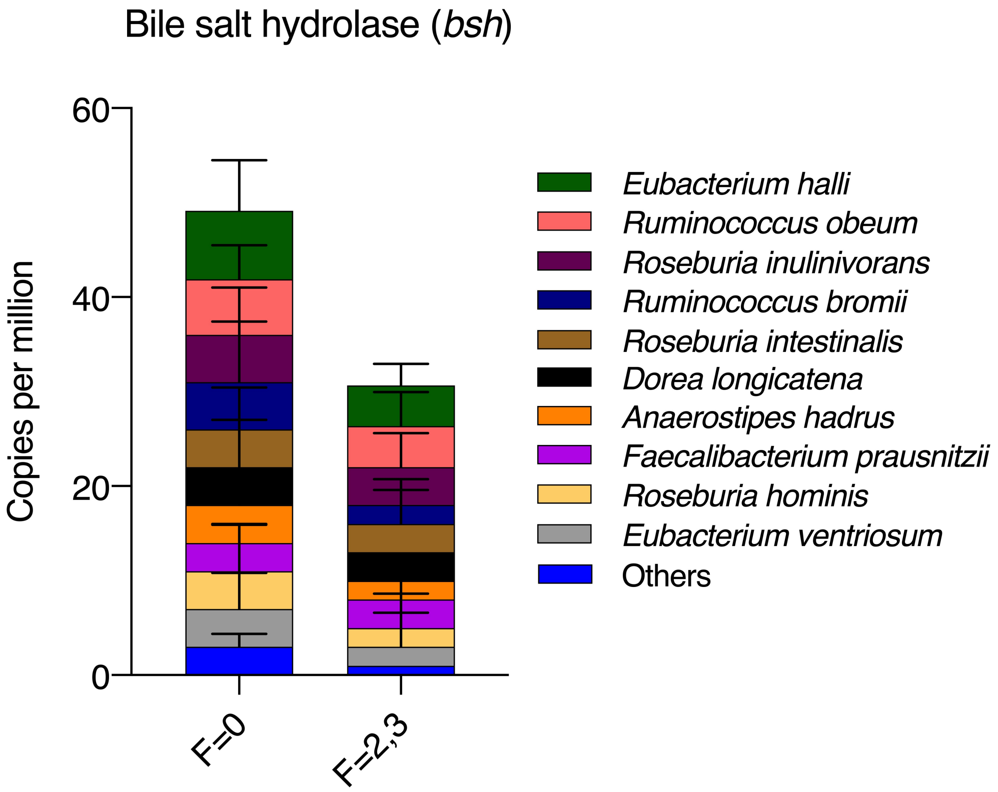

Supplementary Figure 11 Contribution of microbial species to bile salt hydrolase (*bsh*) enzyme. Species contribution was calculated using HUMAnN2 program (F0, n=25; F2–3, n=13). Top 10 contributors are depicted using a stacked bar plot and other contributors are assigned to 'Others'. The stacked bar plots indicate the means with SDs.

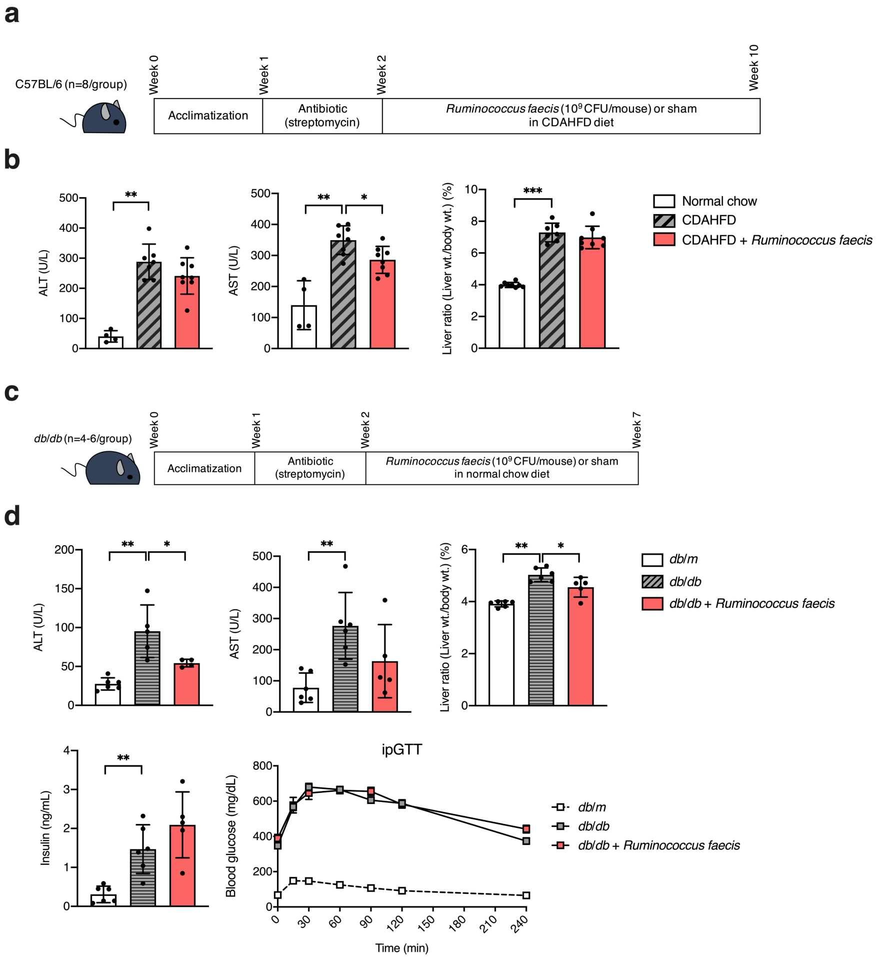

Supplementary Figure 12 Effect of *Ruminococcus faecis* on liver damage using *db/db* and CDAHFD diet-induced NAFLD mouse models. Mice were acclimated for 1 weeks on a standard chow diet. Then, they were treated with streptomycin (1 g/L) in drinking water for colonization of *Ruminococcus faecis*. Following 5 (*db/db*) or 8 (CDAHFD) weeks, mice were given daily 200 µL of either bacteria (10^9 CFU/mouse in PBS) or sham. (a) Scheme of the animal experiment using a CDAHFD-induced NAFLD mouse model. (b) Effects of *Ruminococcus faecis* on serum ALT, AST, and liver ratio (from left, ALT, *P*=0.0061, *n*=4, 7, 8; AST, *P*=0.0040, 0.0225, *n*=4, 8, 8; Liver ratio, *P*=0.0003, *n*=8, 7, 8). (c) Scheme of the animal experiment using a *db/db* mouse. (d) (Top) Effect of *Ruminococcus faecis* on serum ALT, AST, and liver ratio (from left, ALT, *P*=0.0043, 0.0493, *n*=6, 5, 4; AST, *P*=0.0022, *n*=6, 6, 5; Liver ratio, *P*=0.0022, 0.0303, *n*=6, 6, 5; Insulin, *P*=0.0043, *n*=6, 6, 5). (Bottom) Insulin and ipGTT. The bar graphs indicate the

mean with SD. Statistical analysis was performed using the two-sided nonparametric Mann-Whitney test. *$P<0.05$, **$P<0.01$

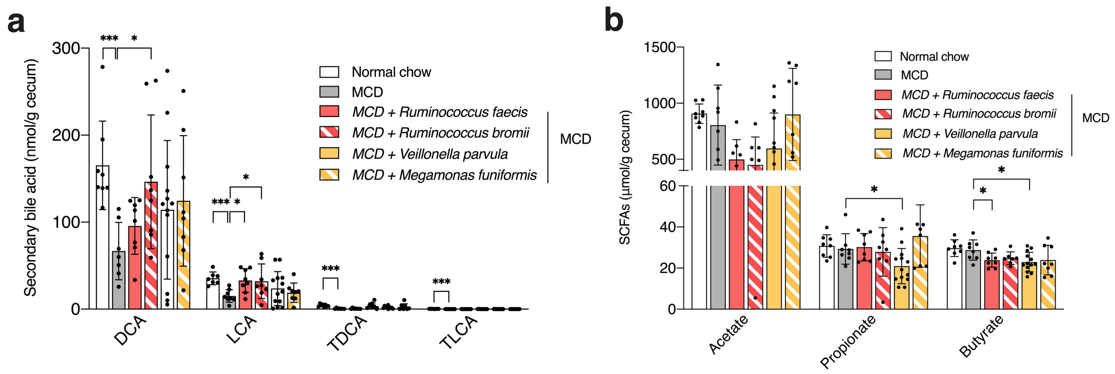

Supplementary Figure 13 Comparison of cecum bile acid or SCFAs profiles of bacteria-treated mice. After bacteria treatment with MCD diet for 5 weeks, bile acid levels were measured in cecal samples and were compared among groups. Absolute value of (a) secondary bile acids (from left, DCA, *P*=0.0003, 0.0205, *n*=8, 7, 8, 8, 13, 8; LCA, *P*=0.0006, 0.0104, 0.0281, *n*=7, 8, 8, 8, 13, 8; TDCA, *P*=0.0003, *n*=8, 7, 7, 8, 13, 8; TLCA, *P*=0.0002, *n*=8, 8, 8, 8, 13, 8) or (b) SCFAs (from left, acetate, *n*=8, 8, 8, 8, 13, 8; propionate, *P*=0.0194, *n*=8, 8, 8, 8, 13, 8; butyrate, *P*=0.0281, 0.0246, *n*=8, 8, 8, 7, 13, 8). The bar graphs indicate the mean with SD. Statistical analysis was performed using the two-sided nonparametric Mann-Whitney test. **P*<0.05, ****P*<0.001

Supplementary Table 1 Baseline characteristics of study subjects stratified by obesity status and histological spectrum of NAFLD.

	**Non-obese (n=64)**				**Obese (n=138)**			
	**No NAFLD**	**NAFL**	**NASH**	***P*-value**	**No NAFLD**	**NAFL**	**NASH**	***P*-value**
N (male/female)	7/14	13/11	7/12		4/6	37/27	24/40	
Age (years)	58.7 ± 10.7	58.3 ± 10.2	60.2 ± 8.84	0.8601 ^{ns}	58 ± 12.6	52.7 ± 14.8	53.6 ± 16.7	0.6463 ^{ns}
BMI (kg/m^2)	22.8 ± 1.67	23.6 ± 1.34	23.6 ± 0.83	0.0871 ^{ns}	27 ± 2.09	28.8 ± 3.25	28.8 ± 3.02	0.1374 ^{ns}
WC (cm)	80 ± 6.53^{a}	82.7 ± 3.45^{ab}	85.4 ± 4b	0.0141 *	92.7 ± 5.93	94.3 ± 8.04	96.6 ± 7.81	0.2328 ^{ns}
AST (IU/L)	31.6 ± 24.4^{a}	28.4 ± 9.05^{b}	54.7 ± 45.7^{bc}	0.002 **	25.6 ± 7.5a	42.3 ± 26.5^{b}	62 ± 32.3^{bc}	< 0.0001 ***
ALT (IU/L)	32.5 ± 32.6^{a}	32.8 ± 17.3^{b}	59 ± 52.9c	< 0.0001 ***	27.8 ± 25.8^{a}	56.9 ± 48.5^{b}	79.1 ± 57.6^{c}	< 0.0001 ***
GGT (IU/L)	44.6 ± 54^{a}	31.8 ± 34.1^{a}	66.7 ± 55.2^{b}	0.0016 **	44.7 ± 51.8^{a}	49.2 ± 57.4^{a}	78.5 ± 79.2^{b}	< 0.0001 ***
HDL cholesterol (mg/dL)	54 ± 14	46.3 ± 10.8	43.8 ± 11.7	0.0527 ^{ns}	58.9 ± 14.3^{a}	47 ± 11.6^{b}	45.5 ± 11.2^{bc}	0.0196 *
LDL cholesterol (mg/dL)	93.5 ± 25.7	111 ± 39.8	97.3 ± 32.1	0.5322 ^{ns}	123 ± 35.8	103 ± 32.3	107 ± 32.7	0.1782 ^{ns}
Albumin (g/dL)	4.1 ± 0.285	4.24 ± 0.257	4.03 ± 0.413	0.1128 ^{ns}	4.12 ± 0.312	4.2 ± 0.254	4.15 ± 0.279	0.516 ^{ns}
Platelet (×10^3/µL)	223 ± 72.2^{ab}	259 ± 54.4^{a}	179 ± 79^{bc}	0.0023 **	232 ± 53	234 ± 59.9	215 ± 69	0.3027 ^{ns}
Ferritin (ng/mL)	103 ± 57.2	109 ± 80.2	173 ± 114	0.1285 ^{ns}	63.8 ± 26.9^{a}	137 ± 88.4^{b}	159 ± 95.1^{bc}	0.0026 **
HA (ng/mL)	66.6 ± 70.2^{ab}	37.1 ± 30.2^{a}	93.9 ± 64.9^{bc}	0.0048 **	76.8 ± 99.2^{ab}	62.1 ± 95.4^{a}	95.7 ± 112^{bc}	0.0344 *
Insulin (µIU/mL)	9.45 ± 3.77^{a}	11.2 ± 5.96^{ab}	13.6 ± 5.85^{b}	0.037 *	11.2 ± 5.55a	18.1 ± 17.1^{ab}	18.2 ± 11.1^{b}	0.0227 *
HbA1c (%)	5.71 ± 0.481^{a}	6.06 ± 0.676^{a}	7.12 ± 1.96^{b}	< 0.0001 ***	5.72 ± 0.326^{a}	6.1 ± 0.814^{ab}	6.6 ± 1.27^{b}	0.0099 **
C-peptide (ng/mL)	1.91 ± 0.61^{a}	2.43 ± 0.832^{ab}	2.88 ± 1.09^{b}	0.0005 ***	2.42 ± 0.916^{a}	4.42 ± 3.4^{b}	4.19 ± 2.39^{bc}	0.0087 **
HOMA-IR	2.56 ± 1.17^{a}	3.12 ± 1.72^{ab}	4.39 ± 2.19^{bc}	0.0085 **	2.92 ± 1.77^{a}	4.96 ± 4.36^{ab}	5.77 ± 4.42^{bc}	0.0131 *
Adipo-IR	4.68 ± 2.85^{a}	6.6 ± 3.59^{ab}	9.63 ± 5.39^{bc}	0.0027 **	6.42 ± 3.28^{a}	10.1 ± 10.1^{ab}	12.8 ± 9.51^{bc}	0.0096 **
FFA (µEq/L)	493 ± 166^{ab}	620 ± 240^{a}	720 ± 268^{bc}	0.0121 *	605 ± 289a	603 ± 259^{ab}	712 ± 238^{bc}	0.0089 **
hsCRP (mg/dL)	0.249 ± 0.428^{a}	0.0896 ± 0.066^{a}	0.354 ± 0.549^{b}	0.0119 *	0.152 ± 0.154^{ab}	0.206 ± 0.403^{a}	0.278 ± 0.336^{bc}	0.0294 *
Cholesterol (mg/dL)	167 ± 28.2	185 ± 41.8	167 ± 42.9	0.3932 ^{ns}	200 ± 43.2	183 ± 34.8	181 ± 40.7	0.4122 ^{ns}
TG (mg/dL)	102 ± 47^{a}	140 ± 44.7^{b}	141 ± 70.9^{ab}	0.0105 *	87 ± 34^{a}	161 ± 82.2^{b}	151 ± 61.9^{bc}	0.0024 **

FPG (mg/dL)	110 ± 25.6	113 ± 28.8	132 ± 42.5	0.0986 ^{ns}	102 ± 14.1	113 ± 27.4	128 ± 55.8	0.2074 ^{ns}
HTN, n (%)	8 (38.1)	8 (33.3)	9 (47.4)	0.641 ^{ns}	4 (40.0)	24 (37.5)	32 (50.0)	0.352 ^{ns}
Diabetes, n (%)	1 (4.76)	8 (33.3)	13 (68.4)	0.0001 ***	1 (10.0)	19 (29.7)	30 (46.9)	0.026 *

Abbreviations: BMI, body mass index; WC, waist circumference; AST, aspartate transaminase; ALT, alanine transaminase; GGT, gamma-glutamyl transferase; NAS, nonalcoholic fatty liver disease activity score; HDL, high-density lipoprotein; LDL, low-density lipoprotein; HA, hyaluronic acid; HbA1c, glycosylated hemoglobin; HOMA-IR, homeostasis model assessment of insulin resistance; Adipo-IR, adipose tissue insulin resistance; FFA, free fatty acid; hsCRP, high-sensitivity C-reactive protein; TG, triglycerides; FPG, fasting plasma glucose; HTN, hypertension. Data are expressed as the mean ± SD or n (%). Mean ± SD or n (%) with different superscript letters indicates significant difference by the nonparametric Kruskal-Wallis test with Dunn's multiple comparison test or the chi-square test. *P<0.05, **P<0.01, ***P<0.001

Supplementary Table 2 Baseline characteristics of study subjects stratified by obesity status and fibrosis severity.

	Non-obese (n=64)				Obese (n=138)			
**Fibrosis stage**	**0**	**1**	**≥ 2**	***P*-value**	**0**	**1**	**≥ 2**	***P*-value**
N (male/female)	27 (11/16)	20 (9/11)	17 (7/10)		25 (17/8)	73 (38/35)	40 (10/30)	
Age (years)	57.67 ± 9.01	57.85 ± 11.80	62.47 ± 8.32	0.2371 ^{ns}	57.08 ± 12.41^{ab}	48.36 ± 15.70^{a}	60.63 ± 13.56^{b}	0.0001 ***
BMI (kg/m^2)	22.81 ± 1.42^{a}	23.76 ± 1.46^{b}	23.71 ± 0.92^{ab}	0.0084 **	27.48 ± 2.58^{a}	29.30 ± 3.20^{b}	28.27 ± 2.97^{ab}	0.0119 *
WC (cm)	79.95 ± 5.2^{a}	83.22 ± 3.53^{ab}	86.33 ± 4.22^{b}	0.0010 **	91.82 ± 6.56	96.16 ± 7.35	95.73 ± 8.98	0.0074 ^{ns}
AST (IU/L)	29.11 ± 21.64^{a}	32.20 ± 10.63^{ab}	56.06 ± 48.13^{b}	0.0017 **	28.40 ± 13.03^{a}	48.99 ± 26.74^{b}	66.13 ± 36.43^{c}	< 0.0001 ***
ALT (IU/L)	31.89 ± 27.83	39.10 ± 30.99	55.71 ± 52.04	0.0886 ^{ns}	38.88 ± 43.67^{a}	70.85 ± 53.02^{bc}	70.85 ± 56.63^{c}	0.0003 ***
GGT (IU/L)	33.6 ± 41.4^{a}	44 ± 46.2^{ab}	69.7 ± 58.3^{b}	0.0046 **	39.6 ± 41^{a}	57.5 ± 56.1^{ab}	85.9 ± 95.2^{b}	0.0016 **
HDL-cholesterol (mg/dL)	51.4 ± 13.4	47.9 ± 11.1	43.1 ± 12.3	0.1701 ^{ns}	48.4 ± 13.1	47.2 ± 12	46.3 ± 11.5	0.9175 ^{ns}
LDL-cholesterol (mg/dL)	104 ± 29.9	108 ± 38.6	90.8 ± 32.3	0.3250 ^{ns}	105 ± 35.7	109 ± 31.7	102 ± 33.8	0.5024 ^{ns}
Albumin (g/dL)	4.14 ± 0.25	4.22 ± 0.29	3.99 ± 0.43	0.1781 ^{ns}	4.12 ± 0.24^{ab}	4.24 ± 0.26^{a}	4.08 ± 0.27^{b}	0.0027 **
Platelet (×10^3/μL)	230.19 ± 48.76^{a}	247.75 ± 74.84^{a}	183.88 ± 95.35^{b}	0.0255 *	238.2 ± 55.38^{a}	241.44 ± 62.49^{a}	188.33 ± 58.05^{b}	0.0001 ***
Ferritin (ng/mL)	117.75 ± 73.97	100.94 ± 74.93	282.19 ± 386.91	0.053 ^{ns}	145.55 ± 89.51	219.37 ± 255.97	169.26 ± 133.32	0.8403 ^{ns}
HA (ng/mL)	33.08 ± 19.62^{a}	64.2 ± 67.47^{a}	109.59 ± 65.56^{b}	0.0002 ***	51.32 ± 66.4^{a}	61.58 ± 90.48^{a}	127.28 ± 129.4^{b}	0.0001 ***
Insulin (μIU/mL)	10.76 ± 5.57	10.05 ± 4.15	13.71 ± 6.19	0.1103 ^{ns}	14.22 ± 11.57^{a}	17.83 ± 15.46^{ab}	19.50 ± 12.58^{b}	0.0148 *
HbA$_{1c}$ (%)	5.86 ± 0.69^{a}	5.98 ± 0.44^{ab}	7.23 ± 2.05^{c}	0.0007 ***	5.87 ± 0.54^{a}	6.15 ± 0.85^{ab}	6.87 ± 1.42^{c}	0.0007 ***
C-peptide (ng/mL)	2.23 ± 0.87^{a}	2.23 ± 0.64^{ab}	2.85 ± 1.17^{bc}	0.0256 *	3.22 ± 1.57^{a}	4.43 ± 3.43^{ab}	4.29 ± 2.28^{bc}	0.0498 *
HOMA-IR	2.94 ± 1.61^{a}	2.81 ± 1.33^{ab}	4.50 ± 2.28^{b}	0.0207 *	3.84 ± 3.35^{a}	4.82 ± 4.18^{ac}	6.69 ± 4.70^{b}	0.0006 ***
Adipo-IR	5.72 ± 3.01	6.33 ± 3.72	9.49 ± 5.95	0.0645 ^{ns}	7.48 ± 6.2^{a}	10.76 ± 9.7^{ab}	13.86 ± 10.55^{bc}	0.0043 **
FFA (μEq/L)	553.96 ± 186.66	615.65 ± 238.13	684.88 ± 308.55	0.2678 ^{ns}	556.08 ± 209.52^{a}	642.76 ± 257.61^{ab}	737.1 ± 259.42^{bc}	0.0059 **
hsCRP (mg/dL)	0.17 ± 0.33^{a}	0.23 ± 0.41^{ab}	0.29 ± 0.48^{bc}	0.0186 *	0.14 ± 0.17^{a}	0.23 ± 0.39^{b}	0.3 ± 0.39^{bc}	0.0121 *
Cholesterol (mg/dL)	177.7 ± 28	180.75 ± 43.68	158.53 ± 44.94	0.1860 ^{ns}	180.96 ± 38.04	188.58 ± 34.01	175.33 ± 44.67	0.2143 ^{ns}
TG (mg/dL)	120.70 ± 45.23	128.42 ± 51.84	137.12 ± 77.04	0.9889 ^{ns}	127.36 ± 51.42	156.23 ± 80.68	155.43 ± 67.19	0.2363 ^{ns}
FPG (mg/dL)	111.15 ± 31.51^{a}	111.85 ± 19.58^{ab}	134.47 ± 43.79^{b}	0.0402 *	110.96 ± 34.36^{a}	107.82 ± 21.43^{a}	144.53 ± 63.53^{b}	0.0001 ***
HTN, n (%)	7 (25.9)	9 (45.0)	9 (52.9)	0.163 ^{ns}	9 (36.0)	30 (41.1)	21 (52.5)	0.357 ^{ns}

Diabetes, n (%)	4 (14.8)	5 (25.0)	13 (76.5)	0.0001 ***	3 (12.0)	23 (31.5)	24 (60.0)	0.0002 ***

Abbreviations: BMI, body mass index, WC, waist circumference; AST, aspartate transaminase; ALT, alanine transaminase; GGT, gamma-glutamyl transferase; HDL, high-density lipoprotein; LDL, low-density lipoprotein; HA, hyaluronic acid; HbA1c, glycosylated hemoglobin; HOMA-IR, homeostasis model assessment of insulin resistance; Adipo-IR, adipose tissue insulin resistance; FFA, free fatty acid; hsCRP, high-sensitivity C-reactive protein; TG, triglycerides; FPG, fasting plasma glucose; HTN, hypertension. Data are expressed as the mean ± SD or n (%). Mean ± SD or n (%) with different superscript letters indicates significant difference by the nonparametric Kruskal-Wallis test with Dunn's multiple comparison test or the chi-square test. *P<0.05, **P<0.01, ***P<0.001

Supplementary Table 3 Histological characteristics of study subjects stratified by obesity status and fibrosis severity.

	Non-obese (n=64)			Obese (n=138)		
**Fibrosis stage**	**0**	**1**	**≥ 2**	**0**	**1**	**≥ 2**
Steatosis, n (%)						
0 (<5%)	15 (55.6)	5 (25.0)	1 (5.9)	7 (28.0)	2 (2.7)	1 (2.5)
1 (5–33%)	7 (25.9)	6 (30.0)	8 (47.1)	12 (48.0)	9 (12.3)	13 (32.5)
2 (34–66%)	4 (14.8)	7 (35.0)	2 (11.8)	3 (12.0)	29 (39.7)	12 (30.0)
3 (>66%)	1 (3.7)	2 (10.0)	6 (35.3)	3 (12.0)	33 (45.2)	14 (35.0)
Lobular inflammation, n (%)						
0	15 (55.6)	3 (15.0)	1 (5.9)	13 (52.0)	5 (6.9)	3 (7.5)
1	12 (44.4)	14 (70.0)	11 (64.7)	12 (48.0)	60 (82.2)	30 (75.0)
2–3	0	3 (15.0)	5 (29.4)	0	8 (11.0)	7 (17.5)
Ballooning, n (%)						
0	22 (81.5)	7 (35.0)	0	22 (88.0)	18 (24.7)	5 (12.5)
1–2	5 (18.5)	13 (65.0)	17 (100.0)	3 (12.0)	55 (75.3)	35 (87.5)
Histological classification, n (%)						
No NAFLD	15 (55.6)	5 (25.0)	1 (5.9)	7 (28.0)	2 (2.7)	0
NAFL	11 (40.7)	13 (65.0)	0	18 (72.0)	40 (54.8)	7 (17.5)
NASH	1 (3.7)	2 (10.0)	16 (94.1)	0	31 (42.5)	33 (82.5)
NAS	1.30 ± 1.46	2.95 ± 1.61	4.00 ± 1.32	1.68 ± 1.22	4.11 ± 1.23	4.08 ± 1.10

Abbreviations: NAFLD, nonalcoholic fatty liver disease; NAFL, nonalcoholic fatty liver; NASH, nonalcoholic steatohepatitis; NAS, NAFLD activity score. Data are expressed as the mean ± SD or n (%).

Supplementary Table 4 Baseline clinical, metabolic, histological, and genetic characteristics of study subjects stratified by obesity status and fibrosis stage.

		Non-obese (n=64)					Obese (n=138)				
**Fibrosis stage**		0	1	2	3	4	0	1	2	3	4
N (male/female)		27 (11/16)	20 (9/11)	9 (5/4)	4 (1/3)	4 (1/3)	25 (17/8)	73 (38/35)	20 (5/15)	7 (2/5)	13 (3/10)
Age (years)		57.7 ± 9.01	57.8 ± 11.8	58.6 ± 9.9	67.2 ± 2.5	66.5 ± 1.73	57.1 ± 12.4	48.4 ± 15.7	55.6 ± 16.4	65.6 ± 8.89	65.8 ± 6.92
BMI		22.8 ± 1.42	23.8 ± 1.46	23.5 ± 0.862	23.9 ± 1.33	24 ± 0.66	27.5 ± 2.58	29.3 ± 3.2	28.5 ± 3.26	28 ± 3.43	28 ± 2.39
WC (cm)		79.9 ± 5.2	83.2 ± 3.53	85.5 ± 2.27	88.1 ± 4.3	86.1 ± 8.37	91.8 ± 6.55	96.2 ± 7.35	95.8 ± 9.02	95.8 ± 13.2	95.6 ± 7.76
SBP (mm Hg)		128 ± 16.9	127 ± 14.3	132 ± 17.1	158 ± 37.4	124 ± 21.7	130 ± 14.8	136 ± 18.4	133 ± 17	132 ± 11.3	126 ± 17.9
DBP (mm Hg)		76.8 ± 12.8	77.4 ± 8.26	80.2 ± 12.9	86.8 ± 19.1	74.5 ± 10.8	80 ± 9.78	84.1 ± 11.8	77.4 ± 13.4	77 ± 10.8	74.3 ± 9.55
AST (IU/L)		29.1 ± 21.6	32.2 ± 10.6	55.1 ± 55.8	80.5 ± 50.1	33.8 ± 8.34	28.4 ± 13	49 ± 26.7	66.6 ± 46.5	70.4 ± 27.9	63 ± 21.7
ALT (IU/L)		31.9 ± 27.8	39.1 ± 31	52.6 ± 53.2	93.5 ± 60.5	25 ± 7.53	38.9 ± 43.7	70.8 ± 53	87.2 ± 74.3	58 ± 19.3	52.6 ± 24.6
GGT (IU/L)		33.6 ± 41.4	44 ± 46.2	69.7 ± 64	373 ± 592	63 ± 43.8	39.6 ± 41	57.5 ± 56.1	79.4 ± 86.1	86.6 ± 68.5	95.6 ± 123
Insulin (μIU/mL)		10.8 ± 5.57	10 ± 4.15	15 ± 6.14	15.3 ± 7.18	9.32 ± 4.29	14.2 ± 11.6	17.8 ± 15.5	21.3 ± 16.1	13.5 ± 4.73	20 ± 8.1
HbA1c (%)		5.86 ± 0.688	5.98 ± 0.445	6.8 ± 0.689	6.05 ± 0.557	9.38 ± 3.52	5.87 ± 0.538	6.15 ± 0.851	7.01 ± 1.45	6.79 ± 1.15	6.74 ± 1.59
HOMA-IR		2.94 ± 1.61	2.81 ± 1.33	4.57 ± 2.05	4.56 ± 2.04	4.27 ± 3.5	3.84 ± 3.36	4.82 ± 4.18	7.28 ± 5.79	4.4 ± 1.42	7.01 ± 3.71
Adipo-IR		5.72 ± 3.01	6.33 ± 3.72	11.1 ± 6.82	8.94 ± 5.18	6.44 ± 4.2	7.48 ± 6.2	10.8 ± 9.7	14.3 ± 12.9	9.29 ± 7.57	15.1 ± 7.42
Diabetes, n (%)		4 (14.8)	5 (25.0)	7 (77.8)	2 (50.0)	4 (100)	3 (12.0)	23 (31.5)	12 (60.0)	4 (57.1)	8 (61.5)
Albumin (g/dL)		4.15 ± 0.259	4.22 ± 0.291	4.19 ± 0.285	3.92 ± 0.45	3.62 ± 0.499	4.12 ± 0.243	4.24 ± 0.26	4.11 ± 0.192	4.06 ± 0.207	4.04 ± 0.393
Platelet (×10^3/μL)		230 ± 48.8	248 ± 74.8	222 ± 67.2	206 ± 123	76.8 ± 32.1	238 ± 55.4	241 ± 62.5	206 ± 50.6	195 ± 48.8	157 ± 63.7
TG (mg/dL)		121 ± 45.2	128 ± 51.8	180 ± 82.9	82.5 ± 26.6	96 ± 31.1	127 ± 51.4	156 ± 80.7	175 ± 71.5	140 ± 48.6	134 ± 64.5
FPG (mg/dL)		111 ± 31.5	112 ± 19.6	123 ± 26.7	123 ± 37	172 ± 66.6	111 ± 34.4	108 ± 21.4	148 ± 78.3	138 ± 30.6	143 ± 53.9
**Histological classification**											
No NAFLD		15 (55.6)	5 (25.0)	0	1 (25.0)	0	7 (28.0)	2 (2.7)	0	0	0
NAFL		11 (40.7)	13 (65.0)	0	0	0	18 (72.0)	40 (54.8)	6 (30.0)	1 (14.3)	0
NASH		1 (3.7)	2 (10.0)	9 (100)	3 (75.0)	4 (100)	0	31 (42.5)	14 (70.0)	6 (85.7)	13 (100)
**Genetic variants**											
PNPLA3 (rs738409)	G/G	6 (22.2)	4 (20.0)	1 (11.1)	0	2 (50.0)	4 (27.4)	20 (27.4)	11 (55.0)	2 (28.6)	6 (46.2)
	C/G	13 (38.1)	13 (65.0)	5 (55.6)	1 (25.0)	2 (50.0)	11 (44.0)	35 (47.9)	4 (20.0)	4 (57.1)	4 (30.8)
	C/C	7 (25.9)	3 (15.0)	2 (22.2)	3 (75.0)	0	7 (17.8)	13 (17.8)	4 (20.0)	1 (14.3)	1 (7.7)
TM6SF2 (rs58542926)	C/C	21 (77.8)	18 (90.0)	6 (66.7)	2 (50.0)	3 (75.0)	18 (72.0)	56 (76.7)	16 (80.0)	4 (57.1)	10 (76.9)
	C/T	5 (18.5)	2 (10.0)	2 (22.2)	2 (50.0)	1 (25.0)	4 (16.0)	11 (15.1)	3 (15.0)	2 (28.6)	1 (7.7)

	T/T	0	0	0	0	0	0	1 (1.4)	0	1 (14.3)	0
MBOAT7-TMC4 (rs641738)	C/C	17 (63.0)	13 (65.0)	5 (55.6)	1 (25.0)	4 (100)	15 (60.0)	42 (57.5)	11 (55.0)	3 (42.9)	7 (53.8)
	C/T	9 (33.3)	5 (25.0)	3 (33.3)	3 (75.0)	0	4 (16.0)	21 (28.8)	8 (40.0)	4 (57.1)	4 (30.8)
	T/T	0	2 (10.0)	0	0	0	3 (12.0)	5 (6.8)	0	0	0
SREBF-2 (rs133291)	C/C	7 (25.9)	6 (30.0)	1 (11.1)	2 (50.0)	2 (50.0)	6 (24.0)	23 (31.5)	8 (40.0)	1 (14.3)	3 (23.1)
	C/T	12 (44.4)	8 (40.0)	6 (66.7)	1 (25.0)	2 (50.0)	7 (28.0)	27 (37.0)	5 (25.0)	5 (71.4)	5 (38.5)
	T/T	3 (11.1)	6 (30.0)	1 (11.1)	1 (25.0)	0	3 (12.0)	8 (11.0)	4 (20.0)	1 (14.3)	2 (15.4)

Abbreviations: BMI, body mass index; WC, waist circumference; SBP, systolic blood pressure; DBP, diastolic blood pressure; AST, aspartate transaminase; ALT, alanine transaminase; GGT, gamma-glutamyl transferase; HbA1c, glycosylated hemoglobin; HOMA-IR, homeostasis model assessment of insulin resistance; Adipo-IR, adipose tissue insulin resistance; TG, triglycerides; FPG, fasting plasma glucose; NAFLD, nonalcoholic fatty liver disease; NASH, nonalcoholic steatohepatitis; PNPLA3, patatin-like phospholipase domain-containing protein 3; TM6SF2, transmembrane 6 superfamily 2; MBOAT7-TMC4, membrane bound O-acyltransferase domain-containing 7 gene and transmembrane channel-like 4 gene; SREBF-2, sterol regulatory element binding transcription factor 2. Data are expressed as the mean ± SD or n (%).

Supplementary Table 5 Medication history of study subjects stratified by obesity status and fibrosis stage.

		Non-obese (n=64)			Obese (n=138)		
**Fibrosis stage**		**0**	**1**	**≥ 2**	**0**	**1**	**≥ 2**
**Diabetes, n**		4	4	13	3	23	24
**Class of medication**	**Agent**						
Biguanide	Metformin	2	4	8	1	16	16
	Vildagliptin	1		1			3
Dipeptidyl peptidase 4 (DPP-IV) inhibitor	Gemigliptin		1	1			3
	Sitagliptin					2	4
	Saxagliptin					1	
	Linagliptin		1	4		2	1
Sulfonylurea (SU)	Glimepiride	1		4	1	5	7
	Gliclazide			1			2
Alpha-glucosidase inhibitor	Acarbose						
Thiazolidinedione (TZD)	Pioglitazone					1	1

Dual or triple combination therapy was also included.

Supplementary Table 6 Baseline characteristics of validation cohort subjects[31] stratified by obesity status and fibrosis severity.

		Advanced fibrosis (BMI<30, n=107)			Advanced fibrosis (BMI≥30, n=61)		
		No	Yes	*P*-value	No	Yes	*P*-value
N (male/female)		91 (23/68)	16 (2/14)		39 (14/25)	22 (9/13)	
Age (years)		45.0 ± 18.6	63.6 ± 14.4	< 0.0001 ***	50.2 ± 13.8	64 ± 11.8	0.0003 ***
BMI (kg/m^2)		24.4 ± 3.1	26.2 ± 2.3	0.0292 *	38.4 ± 12.3	37.0 ± 5.54	0.8087 ^{ns}
Race	White	72	4		16	12	
	Hispanic	17	12		23	10	
	Black	2	0		0	0	

Data are expressed as the mean ± SD or n. Statistical analysis was performed using the two-sided Mann-Whitney test. *P<0.05, ***P<0.001

Supplementary Table 7 Comparison of bile acids levels measured in the cecum of mice.

			Normal chow		MCD	MCD + *R.faecis*		MCD + *R.bromii*		MCD + *V.parvula*		MCD + *M.funiformis*	
		Bile acid	Mean ± SD	*P*-value	Mean ± SD	Mean ± SD	*P*-value	Mean ± SD	*P*-value	Mean ± SD	*P*-value	Mean ± SD	*P*-value
Primary bile acid	Unconjugated	α-MCA	168.9±63.46	**, 0.0093	67.22±58.92	57.1±31.39	0.7789	70.93±45.53	0.5358	94.44±85.51	0.3507	32.64±28.92	0.2319
		β-MCA	264.43±79.64	*, 0.0499	905.83±1029.09	389.03±197.19	0.5737	376.67±164.81	0.4418	783.93±413.19	0.4137	88±93.85	***, 0.0002
		ω-MCA	881.6±257.39	***, 0.0002	132.76±146.57	34.55±30.55	0.281	102.96±73.27	0.7984	47.51±78.5	0.3599	42.41±59.89	0.0939
		CA	35.3±21.36	*, 0.0148	103.13±54.96	100.32±52.37	0.7984	64.73±26.66	0.2786	90.34±52.79	0.6452	32.25±14.11	**, 0.0030
		CDCA	3.03±1.46	0.3823	2.56±2.77	5.5±4.68	0.2786	6.01±6.41	0.1893	2.62±2.76	0.7168	6.02±8.76	0.5054
		UDCA	12.52±3.66	***, 0.0002	3.08±2.13	7.81±7.04	0.3282	4.73±3.29	0.3282	3.68±2.63	0.6452	1.85±1.07	0.1374
	Conjugated	Tα-MCA	9.85±3.18	0.6454	10.89±8.85	8.09±8.69	0.3823	13.38±8.71	0.7209	8.32±6.57	0.5002	13.63±10.9	0.9591
		Tβ-MCA	5.53±2.48	*, 0.0205	11.81±7.81	13.48±6.93	0.2671	22.44±7.78	**, 0.0093	22.56±10	*, 0.0236	19.01±15.71	0.6943
		TCA	7.94±2.72	*, 0.0140	3.9±2.54	3.55±1.89	0.9551	4.25±1.71	0.5358	6.45±3.67	0.1146	1.2±1.07	*, 0.0111
		TUDCA	2.57±0.8	***, 0.0007	0.27±0.05	0.32±0.09	0.2388	0.5±0.18	**, 0.0020	0.51±0.22	*, 0.0101	0.4±0.29	0.5495
Secondary bile acid	Unconjugated	DCA	165.27±50.93	***, 0.0003	66.74±32.92	95.68±32.63	0.0939	146.4±76.75	*, 0.0205	114.17±79.65	0.1348	124.5±75	0.152
		LCA	35.43±7.36	***, 0.0006	15.14±7.28	32.94±13.6	*, 0.0104	32.05±19.91	*, 0.0281	23.74±19.5	0.5466	18.96±11.14	0.4418
	Conjugated	TDCA	4.29±1.47	***, 0.0003	0.63±0.58	2.09±2.07	0.2319	3.52±3.65	0.1893	1.54±1.64	0.3929	2.42±3.67	0.8665
		TLCA	0.31±0.09	***, 0.0002	0.06±0.05	0.19±0.18	0.3899	0.11±0.09	0.4855	0.07±0.07	0.9577	0.02±0.02	0.0684

Abbreviations: CA, cholic acid; CDCA, chenodeoxycholic acid; DCA, deoxycholic acid; LCA lithocholic acid; UDCA, ursodeoxycholic acid; TCA, taurocholic acid; TLCA, taurolithocholic acid; TDCA, taurodeoxycholic acid; TUDCA, tauroursodeoxycholic acid; GCDCA, glycochenodeoxycholic acid; GDCA, glycodeoxycholic acid; GUDCA, glycoursodeoxycholic; GCA, glycocholic acid; α-MCA, α-muricholic acid; β-MCA, β-muricholic acid; ω-MCA, ω-muricholic acid; Tα-MCA, tauro-α-muricholic acid; Tβ-MCA, tauro-β-muricholic acid. Data are expressed as the mean ± SD or n. Statistical analysis was performed between the MCD group and the other groups using the two-sided Mann-Whitney test. *P<0.05, **P<0.05, ***P<0.001

Supplementary References

1 Kashyap, S. *et al.* A sustained increase in plasma free fatty acids impairs insulin secretion in nondiabetic subjects genetically predisposed to develop type 2 diabetes. *Diabetes* **52**, 2461-2474 (2003).

2 Matthews, D. *et al*. Homeostasis model assessment: insulin resistance and β-cell function from fasting plasma glucose and insulin concentrations in man. *Diabetologia* **28**, 412-419 (1985).

3 Sterling, R. K. *et al.* Development of a simple noninvasive index to predict significant fibrosis in patients with HIV/HCV coinfection. *Hepatology* **43**, 1317-1325 (2006).

4 Kim, D. & Kim, W. R. Nonobese fatty liver disease. *Clin. Gastroenterol. Hepatol.* **15**, 474-485 (2017).

5 Koo, B. K. *et al.* Additive effects of PNPLA3 and TM6SF2 on the histological severity of non-alcoholic fatty liver disease. *J. Gastroenterol. Hepatol.* **33**, 1277-1285 (2018).

6 Romeo, S. *et al.* Genetic variation in PNPLA3 confers susceptibility to nonalcoholic fatty liver disease. *Nat. Genet.* **40**, 1461-1465 (2008).

7 Valenti, L. *et al.* Homozygosity for the patatin-like phospholipase-3/adiponutrin I148M polymorphism influences liver fibrosis in patients with nonalcoholic fatty liver disease. *Hepatology* **51**, 1209-1217 (2010).

8 Sookoian, S. & Pirola, C. J. Meta-analysis of the influence of I148M variant of patatin-like phospholipase domain containing 3 gene (PNPLA3) on the susceptibility and histological severity of nonalcoholic fatty liver disease. *Hepatology* **53**, 1883-1894 (2011).

9 Kozlitina, J. *et al.* Exome-wide association study identifies a TM6SF2 variant that confers susceptibility to nonalcoholic fatty liver disease. *Nat. Genet.* **46**, 352-356 (2014).

10 Sookoian, S. *et al.* Genetic variation in transmembrane 6 superfamily member 2 and the risk of nonalcoholic fatty liver disease and histological disease severity. *Hepatology* **61**, 515-525 (2015).

11 Mancina, R. M. *et al.* The MBOAT7-TMC4 variant rs641738 increases risk of nonalcoholic fatty liver disease in individuals of European descent. *Gastroenterology* **150**, 1219-1230 (2016).

12 Luukkonen, P. K. *et al.* The MBOAT7 variant rs641738 alters hepatic phosphatidylinositols and increases severity of non-alcoholic fatty liver disease in humans. *J. Hepatol.* **65**, 1263-1265 (2016).

13 Musso, G., Cassader, M., Bo, S., De Michieli, F. & Gambino, R. Sterol regulatory element-binding factor 2 (SREBF-2) predicts 7-year NAFLD incidence and severity of liver disease and lipoprotein and glucose dysmetabolism. *Diabetes* **62**, 1109-1120 (2013).

14 Caporaso, J. G. *et al.* Global patterns of 16S rRNA diversity at a depth of millions of sequences per sample. *Proc. Natl. Acad. Sci. U. S. A.* **108**, 4516-4522 (2011).

15 Caporaso, J. G. *et al.* QIIME allows analysis of high-throughput community sequencing data. *Nat. Methods* **7**, 335-336 (2010).

16 McDonald, D. *et al.* An improved Greengenes taxonomy with explicit ranks for ecological and evolutionary analyses of bacteria and archaea. *J. ISME* **6**, 610-618 (2012).

17 Caporaso, J. G. *et al.* PyNAST: a flexible tool for aligning sequences to a template alignment. *Bioinformatics* **26**, 266-267 (2010).

18 Cole, J. R. *et al.* The Ribosomal Database Project: improved alignments and new tools for rRNA analysis. *Nucleic Acids Res.* **37**, D141-D145 (2008).

19 Haas, B. J. *et al.* Chimeric 16S rRNA sequence formation and detection in Sanger and 454-pyrosequenced PCR amplicons. *Genome Res.* **21**, 494-504 (2011).

20 Bolger, A. M., Lohse, M. & Usadel, B. Trimmomatic: a flexible trimmer for Illumina sequence data. *Bioinformatics* **30**, 2114-2120 (2014).

21 Langmead, B. & Salzberg, S. L. Fast gapped-read alignment with Bowtie 2. *Nat. Methods* **9**, 357-359 (2012).

22 Truong, D. T. *et al.* MetaPhlAn2 for enhanced metagenomic taxonomic profiling. *Nat. Methods* **12**, 902-903 (2015).

23 Franzosa, E. A. *et al.* Species-level functional profiling of metagenomes and

metatranscriptomes. *Nat. Methods* **15**, 962-968 (2018).

24 Suzek, B. E., Huang, H., McGarvey, P., Mazumder, R. & Wu, C. H. UniRef: comprehensive and non-redundant UniProt reference clusters. *Bioinformatics* **23**, 1282-1288 (2007).

25 Friedman, J. & Alm, E. J. Inferring correlation networks from genomic survey data. *PLoS Comput. Biol.* **8**, e1002687 (2012).

26 Smoot, M. E., Ono, K., Ruscheinski, J., Wang, P. L. & Ideker, T. Cytoscape 2.8: new features for data integration and network visualization. *Bioinformatics* **27**, 431-432 (2010).

27 Buffie, C. G. *et al.* Precision microbiome reconstitution restores bile acid mediated resistance to Clostridium difficile. *Nature* **517**, 205-208 (2015).

28 David, L. A. *et al.* Diet rapidly and reproducibly alters the human gut microbiome. *Nature* **505**, 559-563 (2014).

29 Miquel, S. *et al.* Identification of metabolic signatures linked to anti-inflammatory effects of *Faecalibacterium prausnitzii*. *mBio* **6**, e00300-00315 (2015).

30 Duncan, S. H., Barcenilla, A., Stewart, C. S., Pryde, S. E. & Flint, H. J. Acetate utilization and butyryl coenzyme A (CoA): acetate-CoA transferase in butyrate-producing bacteria from the human large intestine. *Appl. Environ. Microbiol.* **68**, 5186-5190 (2002).

31 Caussy, C. *et al.* A gut microbiome signature for cirrhosis due to nonalcoholic fatty liver disease. *Nat. Commun.* **10**, 1406 (2019).
