# Supplementary material for: Distinct signatures of gut microbiome and metabolites associated with significant fibrosis in non-obese NAFLD
Source: Nat Commun. 2020 Oct 5;11:4982. doi: 10.1038/s41467-020-18754-5 (PMC7536225; doi:10.1038/s41467-020-18754-5)
Supplement: Supplementary file 2 — Reporting Summary [file 41467_2020_18754_MOESM2_ESM.pdf]

nature research

Corresponding author(s): Won Kim and GwangPyo Ko

Last updated by author(s): Aug 27, 2020

Reporting Summary

Nature Research wishes to improve the reproducibility of the work that we publish. This form provides structure for consistency and transparency in reporting. For further information on Nature Research policies, see Authors & Referees and the Editorial Policy Checklist .

Statistics

For all statistical analyses, confirm that the following items are present in the figure legend, table legend, main text, or Methods section.

n/a	Confirmed	
☐	☒	The exact sample size (*n*) for each experimental group/condition, given as a discrete number and unit of measurement
☐	☒	A statement on whether measurements were taken from distinct samples or whether the same sample was measured repeatedly
☐	☒	The statistical test(s) used AND whether they are one- or two-sided<br>*Only common tests should be described solely by name; describe more complex techniques in the Methods section.*
☐	☒	A description of all covariates tested
☐	☒	A description of any assumptions or corrections, such as tests of normality and adjustment for multiple comparisons
☐	☒	A full description of the statistical parameters including central tendency (e.g. means) or other basic estimates (e.g. regression coefficient) AND variation (e.g. standard deviation) or associated estimates of uncertainty (e.g. confidence intervals)
☐	☒	For null hypothesis testing, the test statistic (e.g. *F*, *t*, *r*) with confidence intervals, effect sizes, degrees of freedom and *P* value noted<br>*Give P values as exact values whenever suitable.*
☒	☐	For Bayesian analysis, information on the choice of priors and Markov chain Monte Carlo settings
☒	☐	For hierarchical and complex designs, identification of the appropriate level for tests and full reporting of outcomes
☒	☐	Estimates of effect sizes (e.g. Cohen's *d*, Pearson's *r*), indicating how they were calculated

Our web collection on statistics for biologists contains articles on many of the points above.

Software and code

Policy information about availability of computer code

Data collection	Metagenome data were processed with the open-source bioinformatics pipeline.<br>16S rRNA amplicon sequencing; QIIME (v. 1.8.0), metagenomic shotgun sequencing MetaPhlAn2 (v. 2.6.0), HUMAnN2 (v. 0.11.0)
Data analysis	Microbiome data were analyzed using QIIME (v. 1.8.0), MaAsLin (Galaxy v. 1.0), PyNAST (v. 1.2.2), Cytoscape (v. 3.7.1), ChimeraSlayer (v. microbiomutil-r20110519) MetaPhlAn2 (v. 2.6.0), HUMAnN2 (v. 0.11.0), Bowtie2 (v. 2.3.2), KneadData (v. 0.6.1), Trimmomatic (v. 0.33) and R (v. 3.5.0), with the R packages Vegan (v. 2.5-6) and SparCC (v. 0.1.0). Statistical analyses were carried out using open packages in R, GraphPad Prism (v. 8.4.3), SPSS (v. 25.0), MedCalc (v. 18.2.1). Metabolite data were analyzed with the QuanLynx (v. 4.1) and MassLynx (v. 4.1). The detailed information is described and cited in the Methods and Supplementary Methods sections.

For manuscripts utilizing custom algorithms or software that are central to the research but not yet described in published literature, software must be made available to editors/reviewers. We strongly encourage code deposition in a community repository (e.g. GitHub). See the Nature Research guidelines for submitting code & software for further information.

Data

Policy information about availability of data

All manuscripts must include a data availability statement. This statement should provide the following information, where applicable:

- Accession codes, unique identifiers, or web links for publicly available datasets
- A list of figures that have associated raw data
- A description of any restrictions on data availability

Metagenome sequences from this study were deposited in the European Nucleotide Archive under the study accession number ERP109777 (https://www.ebi.ac.uk/ena/browser/view/PRJEB27662).

Field-specific reporting

Please select the one below that is the best fit for your research. If you are not sure, read the appropriate sections before making your selection.

- [x] Life sciences
- [ ] Behavioural & social sciences
- [ ] Ecological, evolutionary & environmental sciences

For a reference copy of the document with all sections, see nature.com/documents/nr-reporting-summary-flat.pdf

Life sciences study design

All studies must disclose on these points even when the disclosure is negative.

Sample size

1. Human study:
To ensure the statistical power, fecal samples for 16S rDNA amplicon sequencing (n=202) and metagenomic shotgun sequencing (n=38) were obtained and used for analysis. An external NAFLD cohort with 168 subjects available in a public DB was also included as a validation cohort.

2. Mouse intervention study: Animal experiments were sized based on preliminary data and similar studies of other group (Plovier H et al., Nat Med, 2017 and Seo et al., Cell Host Microbe, 2020) to assess the effect of bacteria in mouse models, and expected numbers to ascertain variance in the group and achieve statistical significance in our comparisons.

Data exclusions

The human subjects who met the criteria as belows were excluded from this study.

1. hepatitis B or C virus infection,
2. autoimmune hepatitis, primary biliary cholangitis or primary sclerosing cholangitis,
3. gastrointestinal cancers or hepatocellular carcinoma,
4. drug-induced steatosis or liver injury, 5. Wilson disease or hemochromatosis,
6. excessive alcohol consumption (men: >210 g/week, women: >140 g/week),
7. antibiotics use within the prior month,
8. diagnosis of malignancy within the past year,
9. human immunodeficiency virus infection, and
10. chronic disorders associated with lipodystrophy or immunosuppression.

Replication

1. Human study:
Alterations of specific gut microbiome according to fibrosis severity was observed only in non-obese subjects. These changes were replicated in an independent western cohort.

2. Mouse intervention study:
Animal experiment using MCD diet was performed twice and the results were reproduced successfully. CDAHFD diet model and db/db model were performed once. These two models were added by reviewers' comments in a revision process.

Randomization

The animals were randomly allocated into experimental groups throughout the study.
In human analysis, this is a cross-sectional study of pre-defined cohorts of individuals subdivided by pre-defined diagnosis.

Blinding

For human study, blinding was not applicable during data collection because subjects had already been diagnosed in order to be recruited for the study. The animals were randomly allocated. However, animal histological examination was performed by the technician without any group information.

Reporting for specific materials, systems and methods

We require information from authors about some types of materials, experimental systems and methods used in many studies. Here, indicate whether each material, system or method listed is relevant to your study. If you are not sure if a list item applies to your research, read the appropriate section before selecting a response.

Materials & experimental systems

n/a	Involved in the study	
x		Antibodies
x		Eukaryotic cell lines
x		Palaeontology
	x	Animals and other organisms
	x	Human research participants
x		Clinical data

Methods

n/a	Involved in the study	
x		ChIP-seq
x		Flow cytometry
x		MRI-based neuroimaging

Animals and other organisms

Policy information about studies involving animals; ARRIVE guidelines recommended for reporting animal research

Laboratory animals	The animals used in this study were male C57Bl/6N mice and C57BLKS/J-db/db mice. Six-week-old of male C57BL/6N mice were purchased from Orient Bio (Seongnam, Republic of Korea), and 5 week-old male C57BLKS/J-db/db and control C57BLKS/J-m+/db mice were purchased from SLC (Shizuoka, Japan). The mice were housed in a conventional animal facility according to Seoul national university guidelines. The housing conditions were 12h light and 12h dark cycle, temperature range of 21-24°C and humidity range of 40-50%.
Wild animals	No wild animals were used in the study.
Field-collected samples	No field collected samples were used in the study.
Ethics oversight	All animal experimentation was approved by the Seoul National University Institutional Animal Care and Use Committee (IACUC) (SNU-190117-5-1).

Note that full information on the approval of the study protocol must also be provided in the manuscript.

Human research participants

Policy information about studies involving human research participants

Population characteristics	Boramae NAFLD cohort: total N=202; obesity: 64 non-obese, 138 obese.<br>1. Fibrosis stage: non-obese: 27 (0), 20 (1), 17 (2,3,4); obese: 25 (0), 73 (1), 40 (2,3,4).<br>2. Sex: non-obese: 27 male, 47 female; obese: 65 male, 73 female.<br>3. BMI: non-obese: 23.3 ± 1.38; obese: 28.7 ± 3.1.<br><br>Validation cohort: total N=168; obesity: 107 non-obese, 61 obese.<br>1. Significant fibrosis: non-obese: 16 (yes), 91 (no); obese: 22 (yes), 39 (no).<br>2. Sex: non-obese: 25 male, 82 female; obese: 23 (male), 38 (female).<br>3. BMI: non-obese: 24.7 ± 3.07; obese: 37.9 ± 10.2.
Recruitment	Subjects were from the 'Boramae NAFLD cohort (NCT 02206841)' study at the Seoul Metropolitan Government Seoul National University Boramae Medical Center which is a single center-based, prospective cohort. Validation cohort data was obtained from public dataset reporting microbiome-based biomarkers for predicting NAFLD-related cirrhosis (QIITA Study 11635). Reported results could be biased towards ethnic/regional issues. However, validation using independent Western cohort showed the robustness of results.
Ethics oversight	This study protocol was approved by the Institutional Review Board of Boramae Medical Center (IRB No. 26-2017-48).

Note that full information on the approval of the study protocol must also be provided in the manuscript.
